# A Transcriptome-Wide Analysis Nominates DIO3OS and C9orf139 as Survival-Associated Long Non-Coding RNAs in Glioblastoma

**DOI:** 10.3390/ncrna12050036

**Published:** 2026-09-07

**Authors:** Minseon Kim, Seung-Kyoon Kim, Jaeil Han

**Affiliations:** 1 Department of Microbiology and Molecular Biology, Chungnam National University, Daejeon 34134, Republic of Korea; 2 Department of Convergent Bioscience and Informatic, Chungnam National University, Daejeon 34134, Republic of Korea

**Keywords:** glioblastoma, long non-coding RNA, DIO3OS, C9orf139, overall survival, progression-free interval, MGMT promoter methylation, endpoint definition, optimism correction, external validation

## Abstract

**Background**: Long non-coding RNAs (lncRNAs) are widely proposed as determinants of temozolomide response in glioblastoma (GBM), but many nominations rest on analyst-derived survival endpoints, screens unadjusted for O6-methylguanine-DNA methyltransferase (MGMT) promoter methylation, and models evaluated with the same data used to select them. We asked which lncRNA–outcome associations remain when endpoint definition, confounder adjustment, and external validation are addressed explicitly. **Methods**: We analyzed 94 IDH-wildtype, temozolomide-treated primary GBM patients from The Cancer Genome Atlas (TCGA), using the rule-based progression-free interval (PFI) of the TCGA Pan-Cancer Clinical Data Resource and overall survival (OS) as separate endpoints. A joint transcriptome-wide discovery for OS was performed across TCGA and the identically processed CPTAC-3 cohort (n = 282, 209 deaths), the latter unselected for IDH status because IDH annotation is unavailable for CPTAC-3, with adjustment for MGMT promoter methylation status, cellular composition, and expression subtype. Nominated transcripts were tested for external validation in two independent CGGA cohorts, and incremental discrimination was assessed as the optimism-corrected change in C-index (ΔC-index), benchmarked against the gain expected from a transcriptome-wide search alone. **Results**: No lncRNA was associated with PFI at a transcriptome-wide false-discovery rate (FDR) below 0.05. The joint OS discovery nominated DIO3OS, the only transcript reaching transcriptome-wide significance (hazard ratio 1.38 per standard deviation, 95% confidence interval 1.20–1.58, FDR 0.022), and C9orf139 (FDR 0.065); both were independently significant in each discovery cohort and robust to adjustment for MGMT status, cellular composition, and expression subtype. Both, however, were null with reversed point estimates in the two CGGA cohorts, were unassociated with PFI, and added only modest optimism-corrected discrimination (ΔC-index 0.07–0.11), close to what a transcriptome-wide search alone generates. Replication was thus confined to two cohorts sharing a processing pipeline. **Conclusions**: We nominate DIO3OS and C9orf139 as candidate survival-associated lncRNAs in GBM, requiring prospective evaluation in cohorts with complete IDH annotation and progression endpoints, and show how endpoint definition, selection optimism, and external validation determine which lncRNA associations survive scrutiny.

## 1. Introduction

Glioblastoma (GBM, World Health Organization [WHO] grade 4) is the most common and lethal primary malignant brain tumor in adults, with an annual age-adjusted incidence of approximately 3.2 per 100,000 population in the United States [1]. Despite multimodal treatment with maximal safe surgical resection followed by concurrent radiotherapy and temozolomide (TMZ) chemotherapy, the so-called Stupp protocol [2], the prognosis remains dismal, with a median overall survival (OS) of 14.6 months and a 5-year survival rate below 10% [1,2]. Although several therapeutic advances have entered the clinic over the past two decades, including bevacizumab in the recurrent setting and tumor-treating fields in newly diagnosed disease, none has substantially altered the overall survival trajectory of IDH-wildtype primary GBM. Identifying molecular determinants that explain the substantial inter-patient heterogeneity in outcome therefore remains a central translational priority.

TMZ is a DNA alkylating agent that methylates DNA preferentially at the O6 position of guanine, generating O6-methylguanine adducts that mispair with thymine during replication and trigger futile mismatch–repair cycling, double-strand breaks, and apoptotic cell death [3]. The principal molecular determinant of TMZ activity is O6-methylguanine-DNA methyltransferase (MGMT), a suicide enzyme that directly removes the alkyl adduct and restores DNA integrity. Epigenetic silencing of MGMT by promoter methylation reduces this DNA repair capacity and confers approximately a doubling of median survival under TMZ-containing therapy compared with unmethylated tumors [4]. MGMT promoter methylation is therefore the best-established and most clinically used predictive biomarker for TMZ response in GBM. However, MGMT status is binary while TMZ response is continuous, and a substantial fraction of MGMT-methylated patients fail to respond while a non-trivial subset of MGMT-unmethylated patients derive measurable benefit [3,4].

Long non-coding RNAs (lncRNAs), defined as non-protein-coding transcripts exceeding 200 nucleotides, have emerged as a pervasive layer of gene regulation in human cells [5]. In cancer, lncRNAs have been implicated in tumor initiation, progression, metastasis and drug resistance [6], and one proposed mechanism is that they act as competing endogenous RNAs (ceRNAs) that sequester microRNAs (miRNAs) away from their messenger RNA (mRNA) targets [6,7]. In GBM specifically, several individual lncRNAs, including H19, MALAT1, NEAT1, HOTAIR and CRNDE, have been proposed to modulate TMZ sensitivity through miRNA sponging or other mechanisms [8,9,10,11,12]. ZFPM2-AS1 was reported to promote glioma progression through an SP1/miR-515-5p/SOD2 axis in a cell-line study [13], and LINC00707 has been reported to promote proliferation, invasion and vasculogenic mimicry in glioma cells and to interact with SMAD proteins in TGFβ signaling in other tumor types [14,15,16].

Four recurring methodological weaknesses characterize this literature, and all four can be demonstrated directly in this cohort. First, the survival endpoint is often derived by the analyst from incomplete clinical records rather than taken from a standardized, rule-based resource; in The Cancer Genome Atlas GBM cohort (TCGA-GBM) in particular, progression dates are almost entirely absent, so an analyst-derived “progression-free survival” can silently reduce to overall survival. Second, candidates are nominated in an unadjusted screen and only then tested under adjustment, which shows which pre-selected genes survive adjustment but not which genes of the whole transcriptome are associated with outcome independently of MGMT. Third, discrimination gains are reported as apparent performance in the same patients used to select the candidate, without repeating the selection step inside a resampling procedure. Fourth, ceRNA claims are made from correlation patterns without a matched null and without asking whether the abundances involved could support miRNA titration.

The present work addresses each of these four points explicitly. We analyzed 94 IDH-wildtype, temozolomide-treated primary GBM patients from the TCGA. Because progression dates are absent from the Genomic Data Commons (GDC) clinical records of this cohort, a progression endpoint assembled from those fields, as is common practice, is numerically identical to OS for all 94 patients; we therefore adopted the rule-based progression-free interval (PFI) of the TCGA Pan-Cancer Clinical Data Resource [17] (71 events) as the primary progression endpoint and analyzed OS separately. Candidates were nominated transcriptome-wide under MGMT adjustment rather than re-tested from a pre-selected panel. Because the GDC-harmonized Clinical Proteomic Tumor Analysis Consortium (CPTAC-3) GBM RNA-seq is processed with the same STAR pipeline and GENCODE v36 annotation as the TCGA, the two cohorts could additionally be combined in a joint transcriptome-wide discovery for OS (n = 282, 209 deaths). Incremental discrimination was subjected to bootstrap optimism correction in which the candidate-selection step was repeated inside every resample; external validation was sought in two independent Chinese Glioma Genome Atlas (CGGA) cohorts [18]; and ceRNA claims were evaluated against a matched null and against stoichiometric constraints. The resulting findings, positive and negative, are reported in full. The primary aim of this study is therefore biological and clinical: to identify lncRNAs reproducibly associated with outcome in TMZ-treated GBM and to establish the strength of the evidence behind them. The methodological analyses serve this aim rather than constitute a second one of equal rank: they are the instruments by which credible candidates are separated from artifacts of endpoint definition, selection and confounding. One scope restriction follows from the data: because IDH annotation is unavailable for the CPTAC-3 cohort, the joint discovery cohort is unselected for IDH status, and IDH-wildtype selection is guaranteed only in the TCGA and CGGA cohorts (Section 4.10).

## 2. Results

### 2.1. A Progression Endpoint Derived from GDC Clinical Fields Is Identical to Overall Survival

A progression-free survival endpoint is commonly assembled from the GDC clinical fields as days to recurrence where available, otherwise days to death, otherwise days to last follow-up. Applied to this cohort, that rule produces a variable that is not a progression endpoint at all: days_to_recurrence and progression_or_recurrence are empty for all 94 patients, so the fallback fires for every case and the resulting variable is numerically identical to overall survival (r = 1.000, identical to within 0.1 months in 94 of 94 patients). Any analysis using it will report overall-survival results under a progression label, and will place 84.5% of patients event-free at six months, a figure far more favorable than expected for newly diagnosed IDH-wildtype GBM under chemoradiotherapy and thus a useful warning sign.

**Figure 1:**
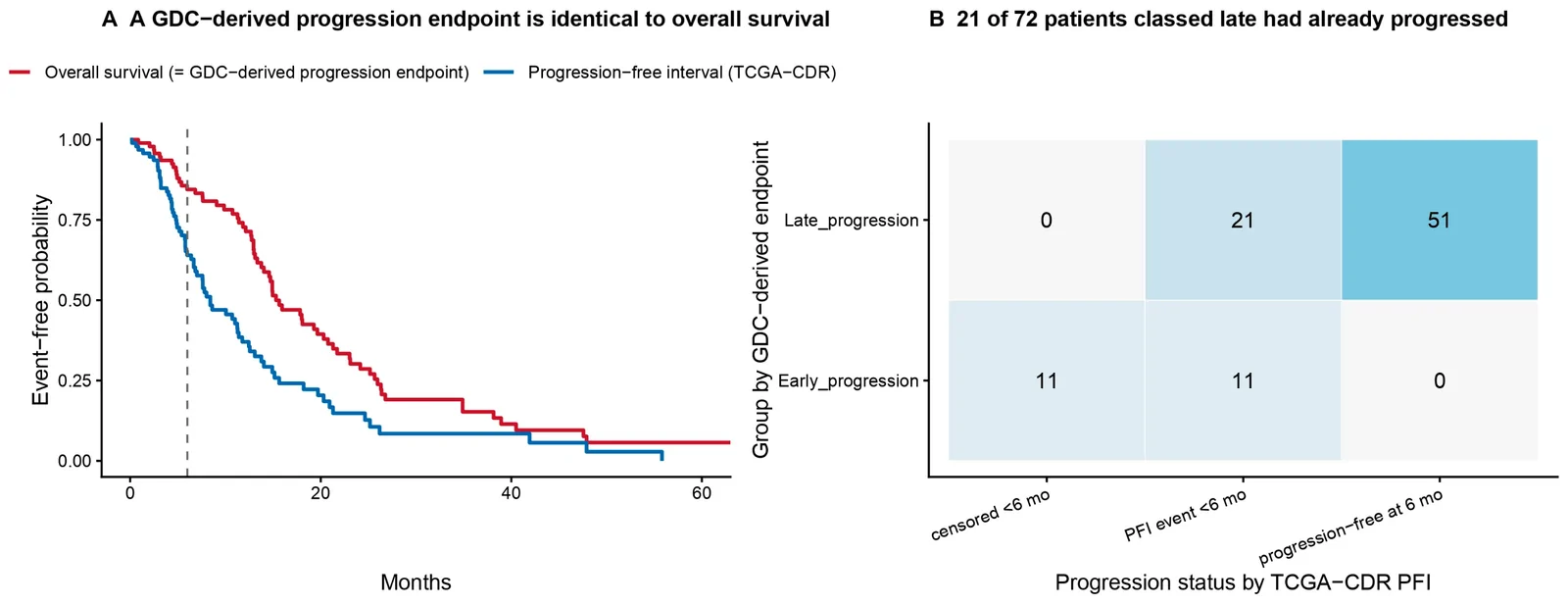
Endpoint definition. (**A**) Kaplan–Meier curves for a progression endpoint derived from the GDC clinical fields, which is identical to overall survival for all 94 patients, and for the TCGA-CDR progression-free interval; the dashed line marks the six-month landmark. (**B**) Cross-classification of six-month group assignment under the GDC-derived endpoint against progression status by PFI.

The rule-based progression-free interval of the TCGA-CDR behaves very differently (Table 1, Figure 1A; Supplementary Table S9). It is available for all 94 patients and records 71 events with a median of 8.4 months, against 68 events and a median of 15.3 months for overall survival; 53 patients have a progression event strictly earlier than their death or censoring time, and the proportion event-free at six months is 64.0%. In the MGMT-known cohort there are 51 PFI events among 69 patients (median PFI 7.8 months; median follow-up among event-free patients 6.9 months) and 50 deaths (median OS 15.0 months; median follow-up among event-free patients 8.5 months). The choice of endpoint also determines group membership: classifying patients at six months on the GDC-derived variable would assign eight patients censored alive before six months to the early-progression group, and would place 21 patients who had already progressed within six months in the late-progression group (Figure 1B). All analyses below use the TCGA-CDR endpoints.

### 2.2. No lncRNA Is Associated with Progression-Free Interval

**Figure 2:**
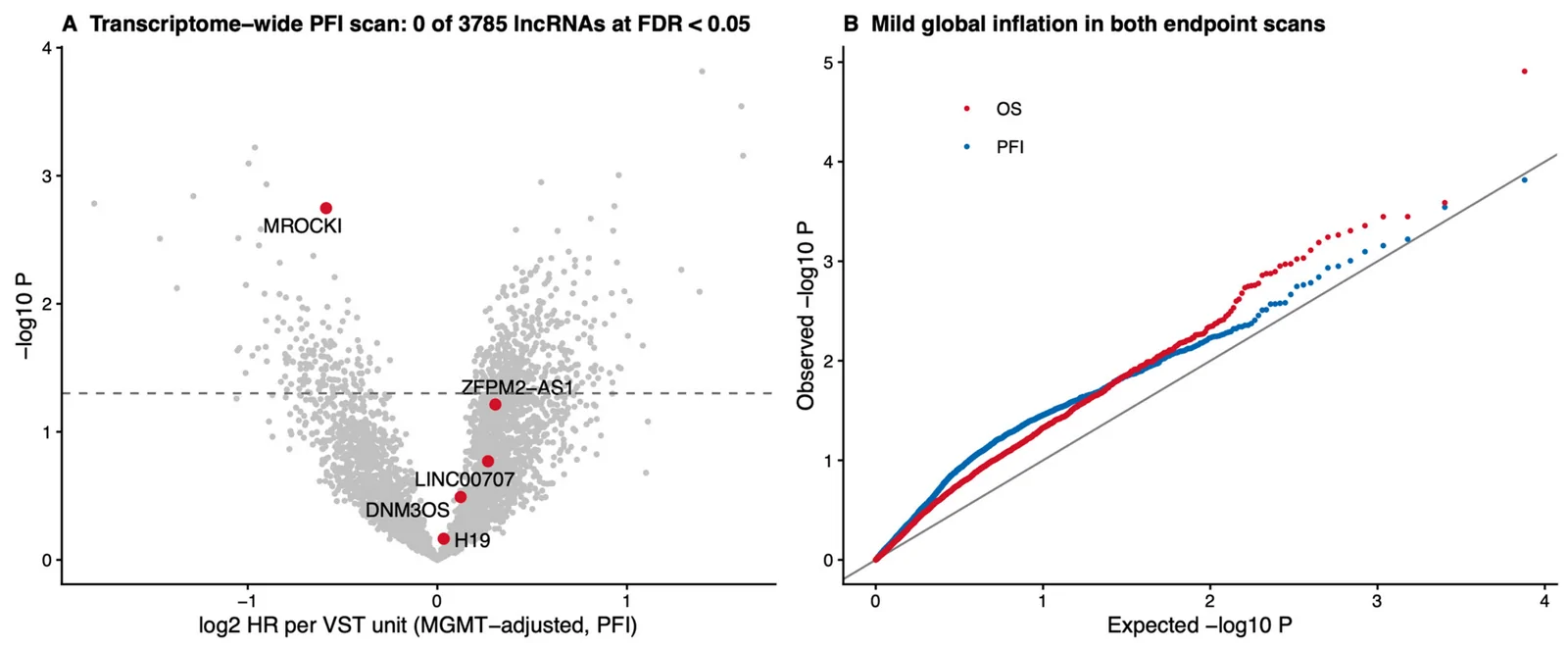
Transcriptome-wide MGMT-adjusted analysis of the progression endpoint. (**A**) Volcano plot across all 3785 expressed lncRNAs (n = 69, 51 events); no transcript reaches FDR < 0.05. (**B**) Quantile–quantile plot of the PFI and OS scans, showing mild global inflation.

Candidate nomination was repeated across the whole lncRNA transcriptome. All 3785 expressed lncRNAs were tested by MGMT-adjusted Cox regression on PFI in the MGMT-known cohort (n = 69, 51 events). No lncRNA reached a transcriptome-wide false-discovery rate below 0.05, or below 0.25 (smallest FDR 0.33; strongest single association AC073210.3, hazard ratio (HR) 2.63 per variance-stabilized unit, 95% confidence interval (CI) 1.60–4.34, *p* = 1.5 × 10^−4^; Figure 2A; Supplementary Table S1). There is mild global inflation, 559 lncRNAs reach a nominal *p* < 0.05 against 189 expected (Figure 2B), so signal is present in aggregate, but no individual transcript is separable from the multiple-testing background at this sample size. This remains our primary finding for the progression endpoint, and it is negative.

### 2.3. A Joint TCGA + CPTAC-3 Discovery Nominates DIO3OS and C9orf139 for Overall Survival

**Figure 3:**
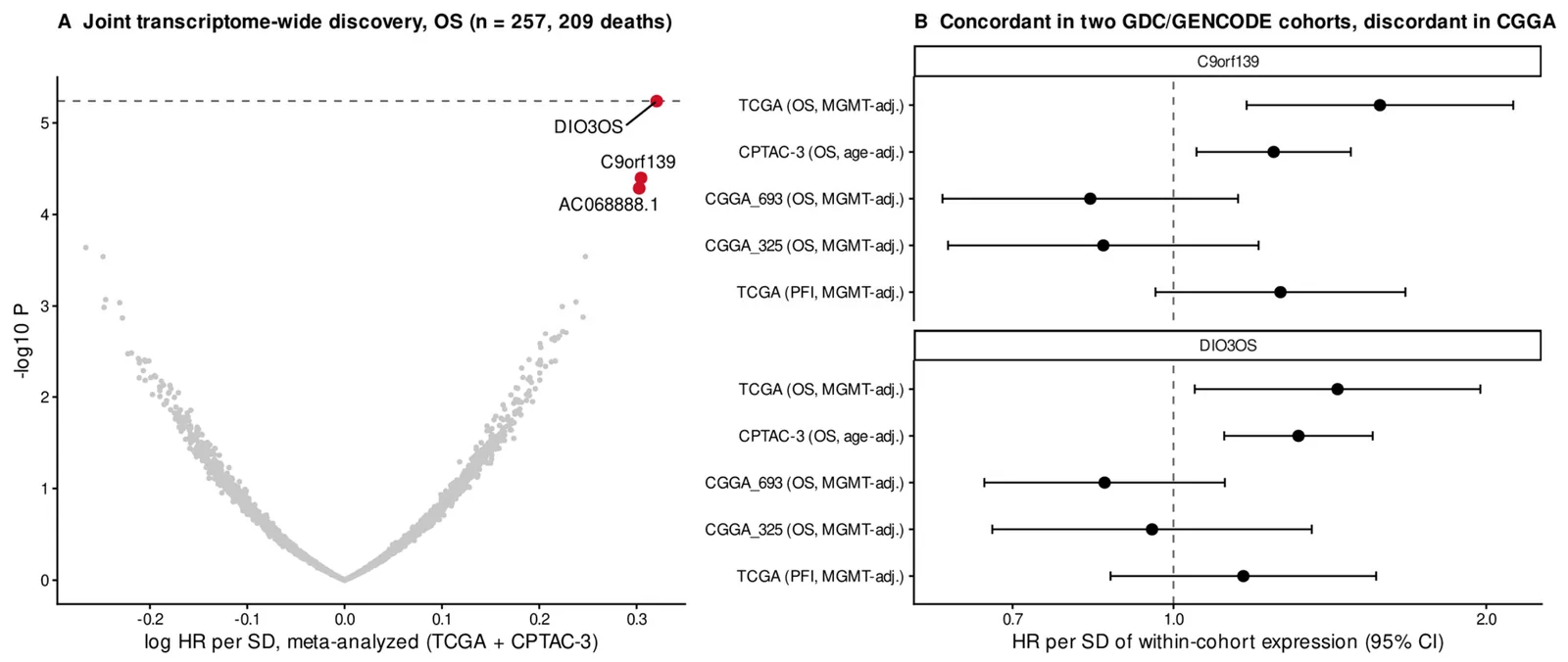
Joint TCGA + CPTAC-3 discovery for overall survival. (**A**) Volcano plot of the inverse-variance meta-analysis across 3784 lncRNAs quantified in both cohorts (n = 282, 209 deaths); the dashed line marks transcriptome-wide FDR 0.05. (**B**) Per-cohort hazard ratios for DIO3OS and C9orf139, showing concordant significance in the two GDC/GENCODE cohorts, null-to-reversed estimates in the two CGGA cohorts, and no association with the progression endpoint.

The limiting factor for the progression analysis is the number of events, and no second cohort records a progression endpoint. For overall survival, however, greater power is available: the GDC-harmonized CPTAC-3 GBM RNA-seq is processed by the same STAR pipeline against the same GENCODE v36 annotation as our discovery data, so the two cohorts share a common gene set (3784 lncRNAs passing the expression filter in both). We therefore analyzed each cohort separately and combined them by inverse-variance meta-analysis, with correction across all 3784 transcripts, giving 282 patients and 209 deaths (Figure 3A; Supplementary Table S12). Because IDH annotation is unavailable for CPTAC-3, this joint discovery cohort is unselected for IDH status (Section 4.10); IDH-wildtype selection applies to its TCGA component and to the within-TCGA analyses below.

DIO3OS was the only transcript to reach transcriptome-wide significance (HR 1.38 per standard deviation, 95% CI 1.20–1.58, *p* = 5.8 × 10^−6^, FDR 0.022), and it was independently significant in each cohort separately, in the same direction (TCGA HR 1.53, *p* = 0.0011; CPTAC-3 HR 1.32, *p* = 0.00097). Two further transcripts reached FDR < 0.10 with the same pattern of independent, concordant significance: C9orf139 (meta HR 1.36, 1.17–1.57, FDR 0.065; TCGA HR 1.68, *p* = 0.00022; CPTAC-3 HR 1.25, *p* = 0.011) and AC068888.1 (meta HR 1.35, FDR 0.065; TCGA *p* = 0.0017; CPTAC-3 *p* = 0.0053). Unlike C9orf139, AC068888.1 did not remain significant under MGMT adjustment (HR 1.34, *p* = 0.076) and was not advanced to further testing. Higher expression was associated with shorter survival in every case. MROCKI, which reaches transcriptome-wide significance for OS in TCGA alone, did not replicate in CPTAC-3 (HR 0.93) and falls well short in the joint analysis (FDR 0.44); we report it as not replicated.

Because these two cohorts share a processing pipeline, their agreement is internal replication across different patients but not replication across independent platforms, and we treat it accordingly throughout.

### 2.4. Both Candidates Are Robust Within TCGA to Adjustment, Influence and Cohort Definition

DIO3OS and C9orf139 were stress-tested in TCGA, where MGMT status, transcriptional subtype and composition scores are available (Table 2; Supplementary Table S13). Both survived adjustment for MGMT status (DIO3OS HR 1.44 per SD, 95% CI 1.05–1.97, *p* = 0.024; C9orf139 HR 1.58, 1.18–2.12, *p* = 0.0024), for marker-based cellular composition in addition to MGMT (DIO3OS 1.76, 1.21–2.57, *p* = 0.0032; C9orf139 1.74, 1.22–2.48, *p* = 0.0022), and for transcriptional subtype (DIO3OS 1.43, *p* = 0.029; C9orf139 1.59, *p* = 0.0030). All patients in the MGMT-known cohort are non-G-CIMP (88 of the 94 patients in the full cohort are annotated non-G-CIMP and the remaining six are unannotated), as expected under IDH-wildtype selection; a test of expression differences by G-CIMP status is therefore not possible in this cohort, and G-CIMP status cannot account for the associations. Neither transcript’s expression differed by expression subtype (*p* = 0.14 and 0.76), so the associations are not a subtype proxy. Proportional-hazards assumptions were satisfied for all DIO3OS and C9orf139 models (scaled Schoenfeld residual *p* = 0.065–0.75; Supplementary Table S13).

Leave-one-patient-out jackknife over all 69 patients showed that neither association depends on a small number of individuals: DIO3OS hazard ratios ranged from 1.36 to 1.57 with *p* between 0.0072 and 0.065 (two of 69 refits crossing 0.05), and C9orf139 from 1.53 to 1.81 with *p* between 0.0012 and 0.0058 (no refit crossing 0.05). Both associations were stronger, not weaker, in the full 94-patient cohort with age, sex and bevacizumab as covariates (DIO3OS HR 1.60, *p* = 0.0005; C9orf139 HR 1.69, *p* = 0.0002). Both transcripts are comfortably expressed rather than at the detection limit (DIO3OS median 20.7 counts per million, interquartile range 11.1–45.4, zero counts in 1.1% of samples; C9orf139 median 105.6, 74.9–154.0, no zero counts), so neither association arises from transcripts measured near the detection limit.

DIO3OS lies within the imprinted DLK1-DIO3 locus at 14q32, which invites a locus-level interpretation, but the data do not support one (Supplementary Table S15). Testing every annotated member of the locus, neither MEG3 (TCGA HR 0.92, *p* = 0.64; CPTAC-3 HR 1.07, *p* = 0.46), nor MEG8 (1.04; 1.02), nor MEG9 (0.99; 1.07) nor MIR381HG (CPTAC-3 1.10, *p* = 0.31) was associated with survival in either cohort, and DIO3OS is only moderately co-expressed with its neighbors (Spearman ρ = 0.35 with MEG3, 0.36 with MEG9, 0.05 with MEG8). The signal is specific to DIO3OS rather than a property of the imprinted locus, and we make no locus-level claim.

### 2.5. Incremental Discrimination Is Modest and Close to What the Search Itself Generates

**Figure 4:**
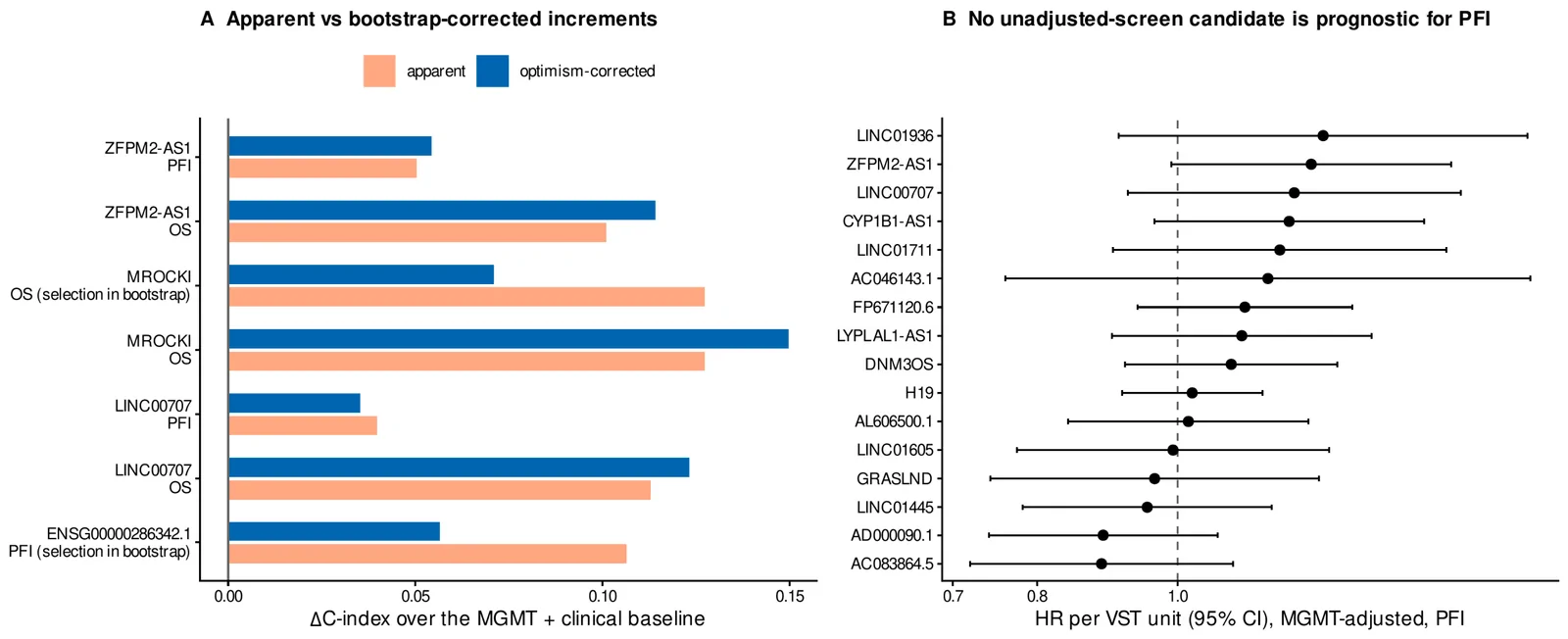
Optimism correction and screen-nominated candidates. (**A**) Apparent versus bootstrap optimism-corrected ΔC-index over the MGMT + clinical baseline, for pre-specified screen candidates on both endpoints and for a transcriptome-wide top-selected transcript with candidate selection repeated inside the resamples (PFI). (**B**) Forest plot of MGMT-adjusted hazard ratios on PFI for the 16 transcripts nominated by the unadjusted screen. Dots mark the hazard ratio point estimate per unit of variance-stabilized expression, horizontal bars the 95% confidence interval, and the dashed vertical line a hazard ratio of 1 (no association).

Over a baseline of MGMT status, age and sex (C = 0.566 for OS), adding DIO3OS raised the C-index to 0.633 (apparent ΔC 0.066) and C9orf139 to 0.669 (apparent ΔC 0.102). Two levels of bootstrap correction must be distinguished, and only the second includes the transcript-selection step. The first treats the transcript as pre-specified and corrects only for overfitting of the fitted model; it leaves these values essentially unchanged (DIO3OS corrected ΔC 0.071; C9orf139 0.113; Supplementary Table S16). Because DIO3OS and C9orf139 were in fact nominated by the transcriptome-wide OS discovery, this pre-specified correction is conditional on the selection already made and should be read as an upper bound, not as a selection-corrected estimate. The second level repeats the entire selection step inside each of 300 bootstrap resamples: all 3785 lncRNAs are re-ranked by MGMT-adjusted Cox *p* value on OS within every resample and the top-ranked transcript is re-selected before the model is refitted. This selection-inclusive analysis, which quantifies what a transcriptome-wide search generates by itself, yields for the OS endpoint an apparent ΔC of 0.127 that corrects to 0.071 (optimism 0.056; the top-selected transcript in the full data being MROCKI), and is reported in Supplementary Table S11 alongside the pre-specified results. This resampling is necessarily confined to the TCGA cohort, in which discrimination is assessed, and therefore benchmarks a TCGA-only search rather than the joint TCGA + CPTAC-3 selection; that it selects MROCKI rather than DIO3OS in the full data is itself an illustration of how unstable top-ranked selection is at this sample size. The same pattern holds on the progression endpoint (baseline C = 0.539): the top-selected transcript gives an apparent ΔC of 0.106 that corrects to 0.056, while the pre-specified screen candidates change little under correction (LINC00707 0.040 apparent to 0.035 corrected; ZFPM2-AS1 0.050 to 0.054; Figure 4A, Supplementary Table S11). Judged against the selection-inclusive benchmark, the incremental discrimination offered by DIO3OS is of the same order as the ceiling the search itself imposes, while C9orf139 sits somewhat above it. We draw the conservative conclusion: neither transcript adds enough discrimination over MGMT and clinical variables to be of predictive use in its own right, and we make no such claim.

### 2.6. External Evidence Is Discordant: Both Candidates Fail in the CGGA

The two CGGA cohorts were held out as a genuinely independent test, on a different population and a different quantification pipeline. Neither candidate replicated, and both point estimates reversed direction: DIO3OS HR 0.86 (95% CI 0.66–1.12, *p* = 0.26) in mRNAseq_693 and 0.95 (0.67–1.36, *p* = 0.79) in mRNAseq_325; C9orf139 HR 0.83 (0.60–1.15, *p* = 0.27) and 0.86 (0.61–1.21, *p* = 0.38) (Figure 3B; Supplementary Table S14). Neither reversal is statistically significant, so the honest description is that the CGGA cohorts are null with point estimates on the opposite side of unity.

We examined whether this could be a technical artifact of the older, RefSeq-style CGGA annotation, which is the reason several screen-nominated transcripts cannot be quantified there at all. This cannot be the explanation. In the CGGA, DIO3OS is coherently co-expressed with its locus neighbors (ρ = 0.59 with MEG3 and 0.51 with MEG9), somewhat more strongly than in the TCGA itself, indicating that the CGGA is measuring the intended transcript competently. The discordance is therefore real and unexplained by annotation. Plausible contributors include population differences between Chinese and North American cohorts, differences in treatment era and post-progression therapy, and the possibility that the concordance between the TCGA and CPTAC-3 partly reflects their shared processing pipeline rather than biology. We cannot distinguish between these with the data available, and we regard the external evidence for both candidates as unresolved.

**Figure 5:**
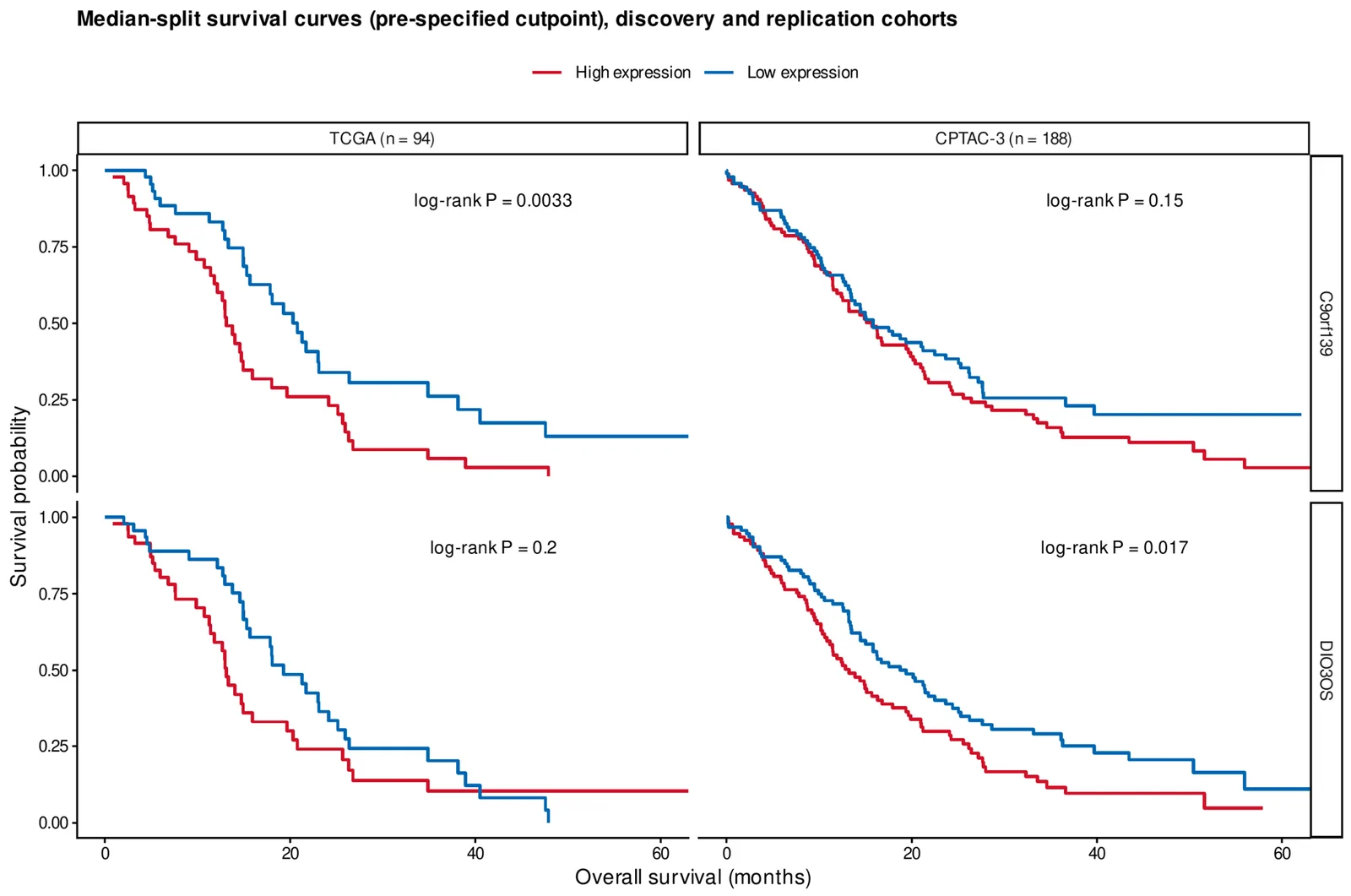
Median-split survival curves for DIO3OS and C9orf139 in the discovery and replication cohorts, using the pre-specified median expression cutpoint (variance-stabilized values: DIO3OS 4.63 in TCGA and 4.10 in CPTAC-3; C9orf139 6.39 and 6.16). Dichotomization loses power relative to the continuous Cox model, which is the inferential test; log-rank *p* values are shown for completeness. Numbers at risk and cumulative events at 0, 6, 12, 18, 24, 36, 48 and 60 months are given in Supplementary Table S18.

Dichotomized visualization reinforces this caution. At the pre-specified median cutpoint, the log-rank test is significant for DIO3OS in CPTAC-3 (*p* = 0.017) but not in the TCGA (*p* = 0.20), and for C9orf139 in the TCGA (*p* = 0.0033) but not in CPTAC-3 (*p* = 0.15) (Figure 5)—the expected loss of power from dichotomization, and a further reason to treat the continuous Cox model as the inferential test and the curves as illustration only.

### 2.7. Candidates from a Conventional Unadjusted Screen Do Not Survive

**Figure 6:**
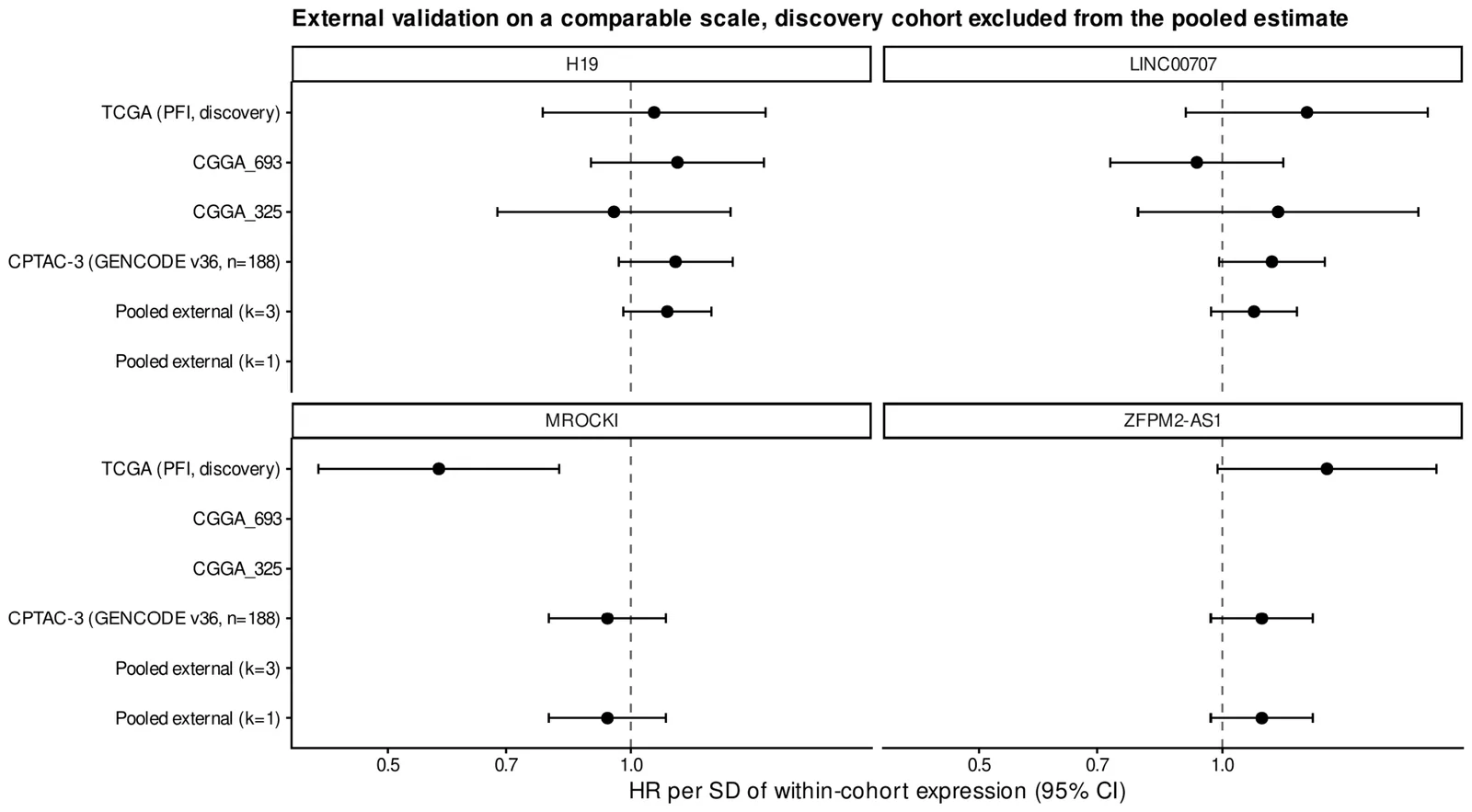
**External validation of the screen-nominated candidates**, per standard deviation of within-cohort expression, with the pooled estimate excluding the discovery cohort. Dots mark the hazard ratio point estimate per standard deviation of within-cohort expression, horizontal bars the 95% confidence interval, and the dashed vertical line a hazard ratio of 1 (no association).

An unadjusted screening design of the kind commonly used to nominate lncRNA candidates (~gender + response group, n = 94) nominates 16 transcripts at |log2 fold change| > 1 and FDR < 0.05. Re-tested on the PFI endpoint under the MGMT-adjusted model, none reaches significance, whether corrected within the panel of 16 or transcriptome-wide (Table 3, Figure 4B; Supplementary Table S10). The strongest are ZFPM2-AS1 (HR 1.24, 95% CI 0.99–1.54, *p* = 0.061; FDR within 16 tests 0.51) and LINC00707 (HR 1.20, 0.92–1.57, *p* = 0.17); H19 is null (HR 1.02, *p* = 0.68), as is DNM3OS (HR 1.09, *p* = 0.32). Substituting the GDC-derived endpoint for PFI turns the same transcripts into apparently strong findings (LINC00707 HR 1.53, ZFPM2-AS1 HR 1.39), which isolates the endpoint as the source of the difference. In the discovery-excluded external analysis these transcripts are also null (LINC00707 pooled HR 1.09, 0.97–1.24; H19 1.11, 0.98–1.26; Table 4, Figure 6).

Before concluding that a candidate cannot be tested in the CGGA, we searched the complete Ensembl cross-reference record of each gene—between 1 and 169 verified identifiers per transcript, including previous symbols, clone-based names and database accessions—against the CGGA gene list. ZFPM2-AS1 (32 identifiers searched), MROCKI (33), LINC01936 (15) and AC083864.5 (ENSG00000287893; 17) are genuinely absent from the CGGA RSEM-genes annotation in both cohorts; H19, LINC00707 and CYP1B1-AS1 are present in both, and DNM3OS only in mRNAseq_325 (Supplementary Table S6). Absence is therefore established by a documented systematic search rather than by a single exact-symbol match. In CPTAC-3, which uses GENCODE v36, all of them are quantified, and all are null on overall survival (LINC00707 HR 1.15 per SD, 95% CI 0.99–1.34; ZFPM2-AS1 1.12, 0.97–1.30; H19 1.14, 0.97–1.34; MROCKI 0.93, 0.79–1.10).

### 2.8. MGMT Adjustment Changes Almost Nothing, and MGMT Confounding Is Not Detectable

It is widely assumed that unadjusted lncRNA–outcome associations in GBM are confounded by MGMT promoter methylation, and that adjustment for MGMT will therefore collapse them. Tested directly in this cohort, that assumption is not supported. Fitting the same genes in the same 69 patients with covariates added one at a time, the addition of MGMT status changed the hazard ratio by at most 3.1% for any candidate (LINC00707 1.17 → 1.20; ZFPM2-AS1 1.21 → 1.22), and adding age, sex and bevacizumab changed estimates by no more than a further 4.7%. Candidate expression did not differ between MGMT strata for any transcript (all FDR ≥ 0.49; H19, an imprinted transcript for which methylation-linked confounding would be most plausible, gave *p* = 0.12 with, if anything, higher expression in unmethylated tumors; Supplementary Table S4). Formal expression × MGMT interaction tests identified no significant interaction after correction (smallest FDR 0.11). With 51 PFI events and roughly 26 per stratum, only a three-fold ratio of hazard ratios is detectable, so opposite-direction stratum-specific effects, which are easily read as evidence of confounding, are within sampling variation here. In this cohort the fragility of screen-nominated candidates is attributable to the endpoint and to cohort-internal selection rather than to MGMT.

### 2.9. Differential Expression Between Six-Month PFI Groups

Genome-wide MGMT-adjusted differential expression between the six-month PFI groups (n = 61 evaluable MGMT-known patients; 25 early, 36 late; eight indeterminate excluded) identified 14 lncRNAs at FDR < 0.05 with |log2 fold change| > 1, including ZFPM2-AS1 (+1.79, FDR 0.020), LINC00707 (+1.73, FDR 0.033) and AC083864.5 (−1.83, FDR 0.047; Supplementary Table S3). Expression differences between outcome groups therefore persist for some screen-nominated transcripts, while their associations with the time-to-event outcome do not. We regard this as a caution rather than a finding: dichotomization discards the timing and censoring information the Cox model uses, and it is the survival analysis that addresses the clinical question.

### 2.10. Candidate Expression Tracks Non-Malignant Compartments of the Bulk Sample

**Figure 7:**
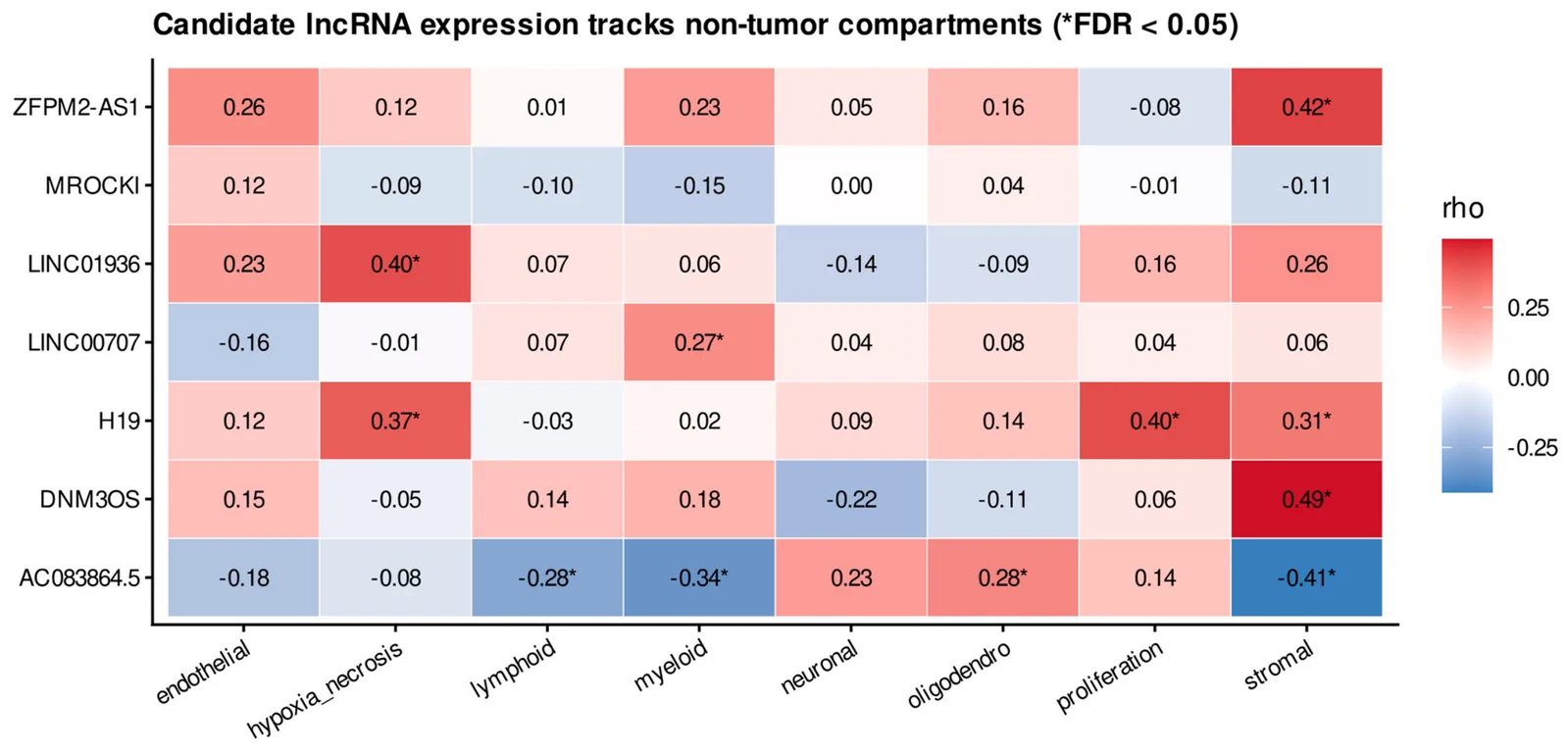
Cellular composition. Spearman correlations between candidate lncRNA expression and marker-based compartment scores; asterisks mark FDR < 0.05.

Marker-based compartment scores show that several screen-nominated transcripts are strongly associated with non-malignant compartments (Figure 7): ZFPM2-AS1 with the stromal score (ρ = 0.42, FDR 7.4 × 10^−4^), DNM3OS with stromal (ρ = 0.49), H19 with proliferation (ρ = 0.40), hypoxia/necrosis (ρ = 0.37) and stromal (ρ = 0.31) scores, LINC01936 with hypoxia/necrosis (ρ = 0.40) and LINC00707 with myeloid content (ρ = 0.27). Adding these scores to the survival models did not abolish the associations that remain, and repeating the full transcriptome-wide scan with composition adjustment again returned no lncRNA for PFI. Composition therefore does not explain the surviving associations, but it does constrain their biological interpretation: transcripts correlating this strongly with stromal, myeloid and hypoxia programs cannot be assumed to be expressed by malignant cells, and absence from normal brain in reference datasets such as GTEx cannot by itself establish tumor-cell-specific expression, since postmortem normal brain and resected tumor differ in cellular composition, RNA quality and processing.

### 2.11. The H19 Correlation Network Against a Matched Null, and Its Stoichiometry

**Figure 8:**
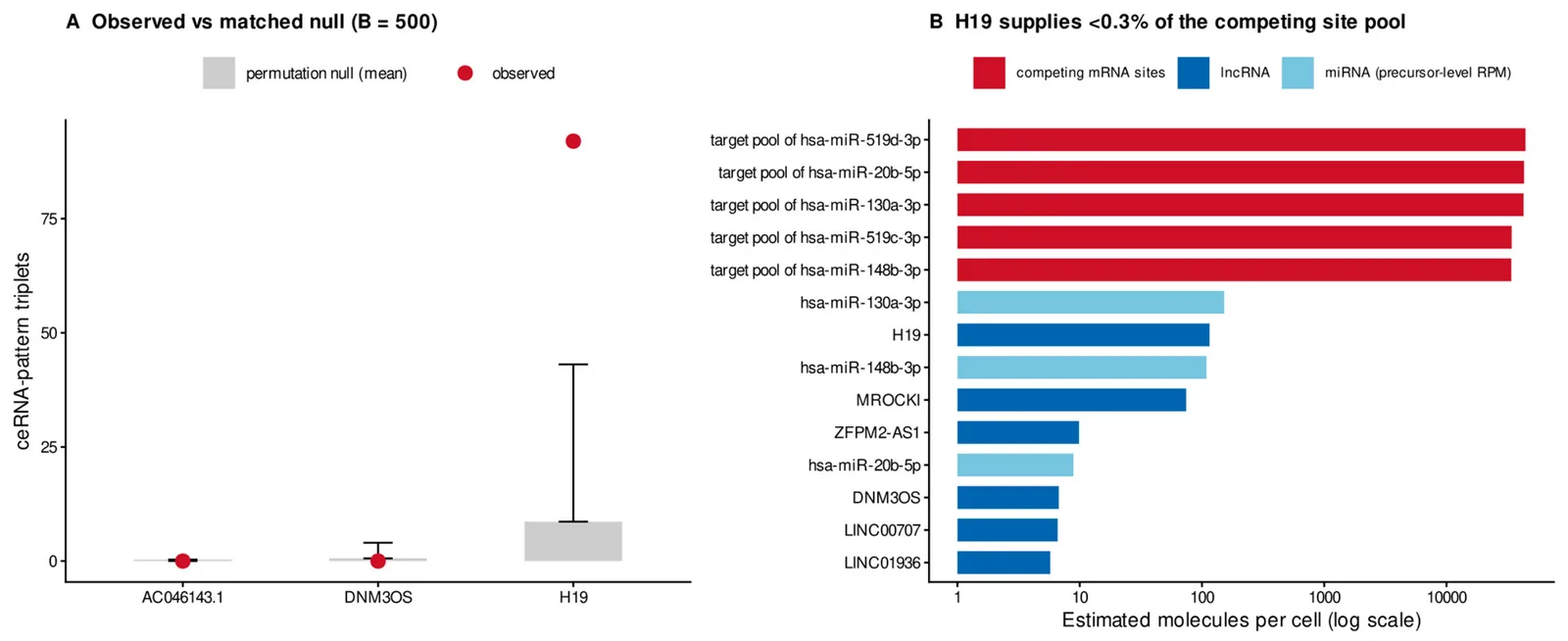
The H19 correlation network against a matched null, and stoichiometry. (**A**) Observed ceRNA-pattern triplet counts against the permutation-matched null (B = 500; bars null means, whiskers 95th percentile). (**B**) Estimated molecules per cell for candidate lncRNAs, implicated miRNAs and competing target-site pools, log scale.

A conventional ENCORI-based analysis of this cohort finds that 117 of 92,062 H19-centered triplets match the canonical ceRNA correlation pattern while 0 of 8429 DNM3OS-centered triplets do, a contrast that invites interpretation as evidence for an H19 mechanism and against a DNM3OS one. Because the number of cataloged miRNAs, targets and testable triplets differs greatly between transcripts, raw counts are not comparable, and we therefore built a permutation-matched null in which each lncRNA’s expression vector was permuted 500 times with the tested triplets and the miRNA–mRNA correlation structure held fixed (Figure 8A; Supplementary Table S7). Within this framework 80,002 H19-centered triplets were evaluable, of which 92 matched the canonical pattern; these exceed a null mean of 8.6, but the null is heavy-tailed (95th percentile 43.1, maximum 249), giving an empirical *p* = 0.022—far weaker than the raw FDR-based statement implies; Fisher’s method, conventionally applied here, is inapplicable because the three correlations share the same samples. For DNM3OS the null predicts 0.59 pattern-matching triplets, so the observed zero is exactly what chance produces (empirical *p* = 1.0) and carries no evidence against a DNM3OS axis.

Stoichiometry gives an independent reason for caution (Figure 8B). H19 is present at a median of about 115 copies per cell, while the competing target-site pools of the implicated miRNAs total roughly 34,000–44,000 transcripts per cell, so H19 contributes on the order of 0.3% of available binding sites, far below the fraction generally considered necessary for effective titration [19,20]. Two of the five contributing miRNAs, hsa-miR-519c-3p and hsa-miR-519d-3p, are essentially undetectable in TCGA-GBM (median 0 reads per million) yet account for 34 of the FDR-significant triplets. We therefore describe an H19-associated expression-correlation network and make no claim of a ceRNA mechanism.

### 2.12. Classical Glioma lncRNAs on the PFI Endpoint

Of 18 widely cited glioma lncRNAs, 14 were detectable. On the PFI endpoint under MGMT adjustment, none reached FDR < 0.05 and only MALAT1 reached a nominal *p* < 0.05 (HR 1.41 per SD, 1.05–1.88, *p* = 0.023, FDR 0.32; Supplementary Table S8). This is not in tension with the experimental literature: knockdown studies ask whether a transcript can influence a phenotype when perturbed, whereas the present analysis asks whether its abundance in bulk tumor tissue predicts outcome across patients.

## 3. Discussion

### 3.1. Principal Findings

The primary question of this study is biological and clinical: which lncRNAs are reproducibly associated with outcome in temozolomide-treated GBM, and how strong is the evidence behind them? On that question, this study has one positive and several negative results. The positive result is that a joint transcriptome-wide analysis of two GENCODE-annotated cohorts nominates DIO3OS, and secondarily C9orf139, as lncRNAs whose higher expression is associated with shorter overall survival in GBM. Both associations are independently significant in each cohort, survive adjustment for MGMT status, cellular composition and transcriptional subtype, are robust to leave-one-patient-out analysis, and strengthen in the full cohort. The negative results are that no lncRNA is associated with the progression-free interval at any defensible threshold; that transcripts nominated by a conventional unadjusted screen, including LINC00707 and ZFPM2-AS1, are not significant on a rule-based progression endpoint and do not replicate externally; that MGMT confounding, the standard explanation for such fragility, is not detectable when tested directly; and that the H19-centered correlation network exceeds a matched null only modestly and is stoichiometrically implausible as a sponge. The methodological analyses reported alongside these results are not a second aim of equal rank; they are the means by which the positive result was separated from artifact. The remainder of this Discussion is organized accordingly: we first weigh the evidence for the two candidates (Section 3.2), then examine why previously nominated candidates do not survive re-examination (Section 3.3), and close with the limitations of the study (Section 3.4).

### 3.2. Strength of Evidence for DIO3OS and C9orf139

We are deliberately conservative about these two transcripts, for five reasons that we state in the abstract rather than leave for a reader to discover. First, both are associated with overall survival and not with progression (DIO3OS PFI *p* = 0.30; C9orf139 *p* = 0.094), and in a uniformly treated cohort an overall-survival association cannot be separated from general prognosis. Second, both are null with reversed point estimates in the two CGGA cohorts, and we could not attribute this to annotation: the CGGA quantifies DIO3OS coherently with its locus neighbors. Third, the two cohorts in which they do replicate share a processing pipeline, so their agreement is weaker evidence than replication across independent platforms would be. Fourth, the incremental discrimination they provide, once optimism is removed, is of the same order as what a transcriptome-wide search generates by chance. Fifth, because the CPTAC-3 cohort lacks IDH annotation, the joint discovery cohort is unselected for IDH status; the IDH-wildtype restriction is certain only within the TCGA, where both candidates were re-tested under MGMT, composition and subtype adjustment. Taken together, these are candidates worth testing prospectively, not biomarkers.

What raises them above the previous candidates is the combination of transcriptome-wide correction, replication in independent patients, robustness to three distinct adjustments and to influence analysis, and adequate abundance. DIO3OS is the antisense transcript at the type 3 deiodinase locus within the imprinted DLK1-DIO3 region, and the null results for MEG3, MEG8, MEG9 and MIR381HG show that the signal is specific to DIO3OS rather than a property of the imprinted domain; we did not test mechanism, and we make no mechanistic claim. C9orf139 is essentially uncharacterized, and we note only that it is well expressed and behaves consistently across our analyses. Both are nominated here as hypotheses for evaluation in cohorts with progression endpoints and independent quantification platforms.

Whether these associations could ever be read as predictive of temozolomide benefit deserves separate comment. Every patient in this cohort received TMZ. Without a comparison group that did not, an association with outcome cannot distinguish an effect specific to TMZ from general prognosis or from response to chemoradiotherapy as a whole; establishing a predictive biomarker would require a treatment-contrasted cohort and a formal treatment × expression interaction test. We examined whether the TCGA could supply such a comparison group and concluded that it cannot: of 284 primary-tumor cases with RNA-seq, 69 are annotated as having received no pharmaceutical therapy, but that group has a median survival of 5.6 months against 13.6 months in the treated group and only 19% received radiotherapy, indicating patients too unwell to be treated or dying before treatment began. Comparing them would introduce confounding by indication and immortal time bias rather than resolve the question. All predictive language has accordingly been removed from this manuscript, and DIO3OS and C9orf139 are described as survival-associated, not as markers of temozolomide response.

### 3.3. Why Previously Nominated Candidates Do Not Survive

Four factors, in order of importance, determined which associations survived in this analysis. Each is common in this literature, and together they explain why candidates nominated by earlier studies, and by the conventional analysis of our own cohort, do not withstand re-examination.

The first is endpoint definition. TCGA-GBM contains essentially no progression dates, so an analyst-derived progression endpoint silently becomes overall survival. Transcripts that appear prognostic on that variable (LINC00707 HR 1.72 per SD for OS, *p* = 0.0013) are null on the rule-based progression endpoint (HR 1.27, *p* = 0.17). Any study reporting progression-free survival in TCGA-GBM without stating the source field and rule set should be read with this in mind, and we recommend the TCGA-CDR [17] as the appropriate source.

The second is selection. Nominating candidates in a screen and evaluating them in the same patients inflates effect sizes and discrimination; repeating the selection inside a bootstrap converts an apparent ΔC of 0.127 into 0.071. Restricting false-discovery correction to a selected panel compounds this: the same *p* values that give an FDR of 0.011 within 16 tests give 0.33 across the transcriptome from which those 16 were drawn.

The third is assuming a confounder rather than testing it. Attributing the fragility of screen-nominated candidates to MGMT confounding is plausible and is the premise of MGMT-aware analytical frameworks. Tested directly here, it fails: adding MGMT moves hazard ratios by at most 3.1%, candidate expression does not differ by MGMT status, and no interaction survives correction. MGMT status should still be modeled explicitly, but in this cohort it was not the source of the fragility. Differential pseudoprogression between MGMT strata [21] would not be removed by covariate adjustment in any case, and with the TCGA-CDR endpoint we cannot distinguish pseudoprogression from true early progression.

The fourth factor is mechanistic rather than prognostic: correlation networks are not evidence of titration. The ceRNA analysis above illustrates a general problem. Triplet counts depend on how many miRNAs and targets a transcript has cataloged, so they cannot be compared between transcripts without a matched null; the three correlations of a triplet are not independent, so combining them by Fisher’s method overstates significance; and neither issue is visible in the output of a standard pipeline. Adding a permutation-matched null and an abundance calculation changes the conclusion from a “statistically robust ceRNA network” to a modest correlation enrichment of uncertain mechanistic meaning. The abundance calculation is the more decisive: at roughly 115 copies per cell against a target-site pool of tens of thousands, H19 cannot plausibly titrate these miRNAs in the average tumor cell [19,20], and whether it does so in a subpopulation is precisely what bulk data cannot address. The downstream genes that emerge—GLUT3 (SLC2A3), HK2, PDK1, XBP1 and CXCL8—belong to broad glycolytic, hypoxia, stress-response and inflammatory programs that covary with necrosis and myeloid content, as the composition analysis confirms, so their appearance is not specific evidence for an H19 axis. GLUT3 in particular is sometimes described as neuron-restricted, but it is expressed in myeloid and other cell types, which is material in a tumor as heavily infiltrated as GBM.

These four factors also delimit how the present null results relate to previous experimental work. LINC00707 has been studied in glioma and other tumors, where it has been associated with proliferation, invasion and vasculogenic mimicry and with TGFβ/SMAD signaling [14,15,16,22]; its clinical prognostic value in a TMZ-treated, MGMT-characterized, IDH-wildtype cohort has not been established, and on a rule-based progression endpoint we cannot support such an association. ZFPM2-AS1 has an established functional model in glioma through the SP1/miR-515-5p/SOD2 axis [13]; our data neither confirm nor refute it, since miR-515-5p is close to undetectable in TCGA-GBM. H19 has repeatedly been linked to TMZ resistance in experimental systems [8]; our finding is only that its abundance in bulk tumor tissue does not predict progression or survival after adjustment. None of these null results falsifies the experimental literature; they delimit the conditions under which lncRNA–outcome associations can be regarded as established in bulk IDH-wildtype primary GBM.

### 3.4. Limitations

The dominant limitation is statistical power: 69 patients with 51 progression events cannot support transcriptome-wide discovery, and our negative progression result should be read as “no lncRNA is detectable at this sample size” rather than as “no lncRNA is associated with progression”. Follow-up is short (median 6.9 months among event-free patients), and the TCGA-CDR endpoint, though rule-based, inherits the incompleteness of the underlying records and cannot separate pseudoprogression from true progression [21]. The CPTAC-3 cohort lacks MGMT status, IDH status and treatment detail, so the joint discovery is adjusted only for age and is unselected for IDH status, and may include a minority of tumors that would not meet the WHO 2021 definition of IDH-wildtype GBM [23]; this is why the candidates were re-tested against MGMT, cellular composition and subtype within the TCGA, and why we describe the joint cohort as glioblastoma unselected for IDH status. Extent of resection is not recorded in the TCGA, and the timing of bevacizumab relative to progression cannot be established. Composition scores are marker-based rather than reference-deconvolved, and no DNA-derived purity estimate is available. The discordance between the GDC-processed cohorts and the CGGA is unresolved. The miRNA data are quantified at the precursor rather than the mature level, and the correlation analysis rests on 43 patients. Finally, the TCGA and CGGA analyses are restricted to IDH-wildtype primary GBM, while the joint discovery concerns GBM unselected for IDH status.

## 4. Materials and Methods

### 4.1. Data Sources, Versions and Access Dates

All resources, versions and access dates are listed in Table 5. RNA-seq gene expression quantification (STAR-Counts) and clinical information for TCGA-GBM were obtained from the Genomic Data Commons (GDC, https://portal.gdc.cancer.gov/; accessed 11 March 2026), generated by the GDC harmonized pipeline using STAR alignment to GRCh38 with GENCODE v36 comprehensive gene annotation. Molecular annotations including IDH mutation status and MGMT promoter methylation status were retrieved from cBioPortal (https://www.cbioportal.org/; accessed 11 March 2026). Standardized survival endpoints were taken from the TCGA Pan-Cancer Clinical Data Resource (TCGA-CDR) [17] (accessed 3 August 2026). Follow-up and new-tumor-event records were re-queried directly from the GDC API on 3 August 2026. CGGA mRNAseq_693 and mRNAseq_325 data [18] were downloaded from http://www.cgga.org.cn (accessed 11 March 2026). CPTAC-3 GBM RNA-seq was obtained from the GDC (STAR-Counts, GENCODE v36; accessed 3 August 2026) and CPTAC GBM proteomic and mature-miRNA data from LinkedOmics [24] (accessed 23 April 2026). GTEx v8 median TPM (accessed 23 April 2026), ENCORI/starBase interactions [25] (accessed 23 April 2026), GDSC2 dose-response data [26] (accessed 23 April 2026) and Ensembl cross-references (release 113 REST API, accessed 3 August 2026) were used as indicated. Each resource is cited in the form requested on its own website.

### 4.2. Cohort Definition

Inclusion criteria were: (i) histologically confirmed primary GBM (WHO grade 4); (ii) IDH-wildtype status per the WHO 2021 classification [23]; (iii) documented TMZ treatment with or without concurrent radiotherapy; and (iv) available RNA-seq and survival data of adequate quality. Recurrent GBM, IDH-mutant tumors and patients without TMZ exposure or without survival information were excluded. The final cohort comprised 94 patients. The primary analytical cohort was the MGMT-known subset (n = 69; 34 MGMT-methylated, 35 MGMT-unmethylated); the 25 patients with MGMT status recorded as unknown were retained only in sensitivity analyses.

### 4.3. Endpoint Definition

A progression-free survival endpoint is commonly derived from the GDC clinical fields as days to recurrence where available, otherwise days to death, otherwise days to last follow-up. In this cohort days_to_recurrence and progression_or_recurrence are empty for all 94 patients, so the fallback applies to every case and the resulting variable is numerically identical to overall survival for 94 of 94 patients (Pearson r = 1.000; Figure 1A). Such an endpoint cannot support progression-specific inference, and we did not use it.

For the present analysis the primary endpoint is the progression-free interval (PFI) of the TCGA-CDR [17], a rule-based endpoint derived centrally from new-tumor-event, recurrence and death records with documented censoring rules. PFI was available for all 94 patients. The time origin is the date of initial pathologic diagnosis; qualifying events are new tumor event (progression, recurrence or new primary) or death from any cause; patients without an event are censored at last contact. Overall survival, defined as death from any cause with censoring at last follow-up, is reported separately as a secondary endpoint and is never pooled with PFI. As an independent check on endpoint completeness, we re-queried the GDC follow-up records: a progression or recurrence flag of “Yes” was present for 54 of 94 patients, a progression date for 36 and a recurrence date for 17, confirming that a valid progression endpoint cannot be assembled from the GDC clinical fields alone and must be taken from the TCGA-CDR.

A binary 6-month classifier was defined on this endpoint for the differential-expression analysis. Patients with a PFI event before 6 months were classified as early progression (n = 32 of 94), patients event-free at 6 months as late progression (n = 51), and patients censored before 6 months without an event were classified as indeterminate and excluded (n = 11) rather than assigned to a group, as they cannot be allocated reliably. For comparison, classifying the same patients on the GDC-derived endpoint would assign 8 patients censored alive before 6 months to the early-progression group and would place 21 patients who had in fact already progressed within 6 months in the late-progression group (Figure 1B). Continuous survival modeling, not the dichotomy, is the primary analysis throughout.

### 4.4. Treatment Covariates

Treatment variables were derived by parsing the GDC all_treatments field (Supplementary Table S17). All 94 patients received radiotherapy (no within-cohort variance), so radiotherapy was not entered as a covariate. Twenty patients (21%) received bevacizumab in addition to TMZ. Extent of resection was not available in the TCGA records and is acknowledged as an unmeasured covariate.

We re-queried the GDC for the timing of each systemic agent relative to the event date. Treatment start and stop days are not populated for individual non-TMZ agents in this cohort, so it is not possible to establish whether bevacizumab was given before or after first progression. Because bevacizumab is most often given at recurrence, an “ever treated” indicator may encode therapy that began after the PFI event and therefore cannot have influenced it. We have consequently removed bevacizumab from the primary model, in which the covariates are MGMT status, age and sex, and report models including bevacizumab only as a sensitivity analysis (Supplementary Table S2). Removing it changed hazard ratios by less than 5% for every candidate.

### 4.5. lncRNA Annotation and Filtering

Genes were classified using GENCODE v36 biotype annotations. lncRNAs were defined using the unified *lncRNA* biotype with historically used subtypes included for compatibility. Low-expression filtering used counts per million (CPM) > 0.5 in at least 30% of samples, retaining 3785 lncRNAs and 14,104 protein-coding genes.

### 4.6. Transcriptome-Wide, MGMT-Adjusted Nomination

Candidates are nominated transcriptome-wide, not from a pre-selected panel. All 3785 expressed lncRNAs were tested individually in a multivariable Cox model, Surv (time, event)~expression + MGMT status + age + sex, in the MGMT-known cohort (n = 69), with expression on the variance-stabilizing-transformed (VST) scale, separately for PFI and for OS. Benjamini–Hochberg correction was applied across all 3785 tests. In parallel, differential expression between the corrected 6-month groups was assessed genome-wide with DESeq2 v1.42 [27] using the design~MGMT_status + age_years + gender + pfi_group6 in the evaluable MGMT-known patients (n = 61; 25 early, 36 late), with Benjamini–Hochberg correction across all tested genes. For comparison with conventional practice, we also ran an unadjusted screening design (~gender + response group, n = 94) of the kind commonly used to nominate lncRNA candidates; the 16 transcripts it nominates are re-tested under the full model and reported in full.

### 4.7. Isolating the MGMT Term and Testing for Interaction

To isolate the contribution of MGMT adjustment specifically, the same genes were fitted in the same patients with covariates added one at a time (expression alone; + MGMT; + age and sex; + bevacizumab), so that any change is attributable to the added term rather than to a change in cohort or design. The relationship between candidate expression and MGMT status was tested directly by Wilcoxon rank-sum test. Rather than assigning candidates to descriptive robustness tiers, effect modification was assessed by formal expression × MGMT interaction tests (Wald test on the interaction term, with a likelihood-ratio test of the nested models), reported together with an explicit statement of the interaction size the cohort can detect.

### 4.8. Optimism-Corrected Discrimination

Incremental discrimination over a baseline model of MGMT status, age and sex was quantified by the difference in Harrell’s C-index (ΔC), computed with survcomp v1.52.0 [28]. Because both the candidate and the model were derived in the same 69 patients, apparent ΔC is optimistically biased. We therefore performed bootstrap optimism correction with 300 resamples in which the entire candidate-selection step was repeated inside each resample: in every bootstrap sample all 3785 lncRNAs were re-ranked by MGMT-adjusted Cox *p* value, the top-ranked gene was re-selected, the model was refitted, and the difference between its performance in the bootstrap sample and in the original sample was averaged to estimate optimism. Pre-specified single genes were bootstrapped in the same way without the selection step, for comparison.

### 4.9. Cellular Composition of Bulk Tumor Samples

Bulk GBM expression reflects tumor purity and infiltrating non-malignant cells. Compartment scores were computed as the mean of z-scored, VST-transformed expression of curated marker sets for myeloid (PTPRC, CD68, AIF1, CSF1R, ITGAM, TYROBP, FCER1G, C1QA, C1QB, CD163, MRC1), lymphoid, endothelial, stromal, neuronal, oligodendroglial, proliferation and hypoxia/necrosis programs (full marker lists in Supplementary Table S5). These are marker-based compartment scores and are not equivalent to reference-based deconvolution or to DNA-derived purity estimates, which are not available for this cohort; they are used to ask whether candidate associations are explained by composition. Candidate expression was correlated with each score (Spearman), and all survival models were refitted with the myeloid, neuronal, stromal, proliferation and hypoxia scores added as covariates, including a repeat of the full transcriptome-wide scan.

### 4.10. External Validation

Two independent cohorts were used, and the discovery cohort is excluded from the estimate we present as validation.

First, CGGA mRNAseq_693 and mRNAseq_325 [18] were filtered with the same criteria as the TCGA (IDH-wildtype primary GBM, TMZ-treated, MGMT status known), yielding n = 76 and n = 52. Before concluding that any candidate is unavailable in the CGGA, we performed a systematic identifier search: for each candidate the complete Ensembl cross-reference record (all external databases and their synonym lists, 1 to 169 identifiers per gene) was retrieved from the Ensembl REST API and every identifier was matched against the CGGA gene list. Only identifiers verified in this way were used; no clone-based or previous-symbol match was accepted on the basis of resemblance alone.

Second, because the CPTAC RNA-seq matrix distributed through LinkedOmics is protein-coding-focused (19,006 gene symbols, of which only 2 are LINC identifiers) and therefore cannot quantify lncRNA candidates, we assembled a further external cohort from the GDC-harmonized CPTAC-3 GBM RNA-seq, which uses STAR v2.7.5c with GENCODE v36, the same annotation as our discovery data. Of 200 primary-tumor cases with STAR-Counts data, 188 with a primary diagnosis of glioblastoma, known vital status and survival time were analyzed (141 deaths; median OS 13.4 months). The GDC does not expose MGMT status, IDH status, sex or interpretable treatment detail for this project, so the CPTAC-3 model is Surv (OS)~expression + age; this cohort therefore provides an unadjusted external test of direction and magnitude, not a like-for-like replication, and is presented as such. In particular, because IDH status cannot be established from the GDC records for CPTAC-3, this cohort is unselected for IDH status and may include a minority of tumors that would not meet the WHO 2021 definition of IDH-wildtype GBM [23]; the joint discovery cohort is therefore described throughout as glioblastoma unselected for IDH status, and IDH-wildtype-restricted inference is confined to the TCGA and CGGA cohorts.

To make hazard ratios comparable across datasets with different quantification scales (VST for TCGA and CPTAC-3, log2(RSEM + 1) for CGGA), expression was z-scored within each cohort and hazard ratios are reported per standard deviation. Cohort estimates were combined by inverse-variance fixed-effect meta-analysis on log(HR) with I2 reported; the primary pooled estimate excludes the discovery cohort.

### 4.11. Correlation-Based ceRNA Analysis, Matched Null and Stoichiometry

A conventional ENCORI-based triplet analysis was performed and interpreted against a matched null. For each candidate lncRNA, mature miRNAs from ENCORI [25] were mapped to TCGA precursor identifiers and three Spearman correlations were computed (lncRNA–miRNA, miRNA–mRNA, lncRNA–mRNA) in the 43 patients with all three data layers and known MGMT status. Because the number of cataloged miRNAs, targets and testable triplets differs greatly between lncRNAs, raw counts of pattern-matching triplets cannot be compared across genes. We therefore constructed a permutation-matched null in which the lncRNA expression vector was permuted across the 43 patients 500 times while the identity of every tested triplet and the entire miRNA–mRNA correlation structure were held fixed, and the number of pattern-matching triplets was recounted in each permutation. Empirical *p* values are the proportion of permutations reaching the observed count. Because the three correlations of a triplet are not independent, Fisher’s combined *p*, commonly used for this purpose, is not an appropriate joint test and overstates significance; the permutation null replaces it.

Stoichiometric plausibility was assessed by converting abundances to approximate molecules per cell, assuming 2 × 10^5^ mRNA and 1 × 10^5^ miRNA molecules per cell (assumptions stated explicitly, as absolute quantification is not available for these samples): lncRNA and mRNA transcripts per million were scaled by the mRNA total, and miRNA reads per million by the miRNA total. The competing target-site pool for each miRNA was estimated as the summed median abundance of all cataloged target mRNAs of that miRNA expressed in the cohort.

### 4.12. Joint Transcriptome-Wide Discovery Across the TCGA and CPTAC-3

Because TCGA-GBM and the GDC-harmonized CPTAC-3 GBM RNA-seq are processed by the same STAR pipeline against GENCODE v36, the two cohorts can be analyzed on a common gene set; 3784 lncRNAs passed the expression filter in both. Each cohort was analyzed separately (both age-adjusted, with expression z-scored within cohort), and the two log hazard ratios were combined by inverse-variance fixed-effect meta-analysis, with Benjamini–Hochberg correction across all 3784 transcripts. Because the two cohorts contain different patients, a transcript reaching significance in each individually constitutes internal replication; because they share a processing pipeline, this is not equivalent to replication across independent platforms, and we state that limitation explicitly. Transcripts nominated in this way were then stress-tested in the TCGA against MGMT status, marker-based composition scores and transcriptional subtype, by leave-one-patient-out jackknife over all 69 patients, and in the full 94-patient cohort; and were tested in the two CGGA cohorts as a held-out set. Kaplan–Meier curves use the pre-specified median expression cutpoint and are presented as visualization only, the continuous Cox model being the inferential test.

### 4.13. Statistical Analysis

All analyses were performed in R v4.3 with set.seed (42). Multiple-testing correction used the Benjamini–Hochberg method throughout. A two-sided *p* < 0.05 was considered significant unless otherwise noted. Proportional-hazards assumptions were checked by scaled Schoenfeld residuals for all reported models. All analysis code, the clinical-derivation rules, sample identifiers and model output tables are deposited publicly (Section “Data Availability Statement”); nothing is available “on request” only.

## 5. Conclusions

An endpoint-corrected, transcriptome-wide analysis of glioblastoma nominates DIO3OS, and secondarily C9orf139, as lncRNAs whose expression is associated with shorter overall survival in two independent GENCODE-annotated cohorts—the TCGA cohort IDH-wildtype by selection, the CPTAC-3 cohort unselected for IDH status—robustly to adjustment for MGMT status, cellular composition and transcriptional subtype. Neither is associated with progression, neither replicates in two CGGA cohorts, and neither adds discrimination beyond what a transcriptome-wide search generates by chance; they are therefore candidates for prospective evaluation rather than biomarkers. In the same analysis, no lncRNA is associated with progression-free interval, transcripts nominated by a conventional unadjusted screen do not survive, and the MGMT-confounding explanation for such fragility is not supported when tested directly. We report both the positive and the negative findings, together with the framework that produced them, because the pitfalls they expose—an analyst-derived endpoint that reduces to overall survival, nomination and evaluation in the same data, an assumed confounder that is never tested, and mechanism claimed from correlation alone—are readily encountered in this literature and are not visible in the output of a standard pipeline.

## Figures and Tables

**Table 1:** Endpoint audit. A progression endpoint derived from the GDC clinical fields is identical to overall survival; the TCGA-CDR progression-free interval differs substantially from both.

Endpoint	n	Events	Censored	Median (Months)	Censored < 6 Months	Events < 6 Months	Event-Free at 6 Months
Progression endpoint derived from GDC fields	94	68	26	15.3	8	14	84.5%
Overall survival (rebuilt from GDC)	94	68	26	15.3	8	14	84.5%
Overall survival (TCGA-CDR)	94	68	26	15.3	8	14	84.5%
Progression-free interval (TCGA-CDR)	94	71	23	8.4	11	32	64.0%
Overall survival, MGMT-known cohort	69	50	19	15.0	5	14	79.2%
Progression-free interval, MGMT-known cohort	69	51	18	7.8	8	25	62.0%

**Table 2:** DIO3OS and C9orf139: joint discovery, robustness within TCGA, and held-out external testing. Hazard ratios are per standard deviation of within-cohort expression.

Analysis	Cohort/Model	DIO3OS	C9orf139
Joint discovery (OS)	TCGA + CPTAC-3 meta, n = 282, 209 deaths	1.38 (1.20–1.58), FDR 0.022	1.36 (1.17–1.57), FDR 0.065
Discovery cohort alone	TCGA, age-adjusted	1.53, *p* = 0.0011	1.68, *p* = 0.00022
Replication cohort alone	CPTAC-3, age-adjusted	1.32, *p* = 0.00097	1.25, *p* = 0.011
MGMT-adjusted	TCGA, n = 69, OS	1.44 (1.05–1.97), *p* = 0.024	1.58 (1.18–2.12), *p* = 0.0024
MGMT + composition-adjusted	TCGA, n = 69, OS	1.76 (1.21–2.57), *p* = 0.0032	1.74 (1.22–2.48), *p* = 0.0022
MGMT + subtype-adjusted	TCGA, n = 69, OS	1.43 (1.04–1.96), *p* = 0.029	1.59 (1.17–2.15), *p* = 0.0030
Full cohort	TCGA, n = 94, age + sex + bevacizumab	1.60, *p* = 0.0005	1.69, *p* = 0.0002
Progression endpoint	TCGA, n = 69, PFI, MGMT-adjusted	1.17 (0.87–1.57), *p* = 0.30	1.27 (0.96–1.67), *p* = 0.094
Jackknife (69 refits)	TCGA, OS, MGMT-adjusted	HR 1.36–1.57; 2 of 69 refits *p* > 0.05	HR 1.53–1.81; 0 of 69 refits *p* > 0.05
Held-out external	CGGA_693, OS, MGMT-adjusted	0.86 (0.66–1.12), *p* = 0.26	0.83 (0.60–1.15), *p* = 0.27
Held-out external	CGGA_325, OS, MGMT-adjusted	0.95 (0.67–1.36), *p* = 0.79	0.86 (0.61–1.21), *p* = 0.38
Discrimination over MGMT + clinical (transcript treated as pre-specified)	TCGA, OS (baseline C = 0.566)	ΔC 0.066 apparent, 0.071 corrected	ΔC 0.102 apparent, 0.113 corrected
Abundance	TCGA, counts per million	median 20.7 (IQR 11.1–45.4), 1.1% zeros	median 105.6 (74.9–154.0), 0% zeros

**Table 3:** The 16 lncRNAs nominated by the unadjusted screening design, re-tested under the MGMT-adjusted model on the progression-free interval (n = 69, 51 events). AC083864.5 (ENSG00000287893) and AC046143.1 (ENSG00000229334) are distinct transcripts.

lncRNA	HR per VST Unit	95% CI	*p*	FDR Within 16 Tests	Transcriptome-Wide FDR	HR Reported Previously (Mislabeled Endpoint)
ZFPM2-AS1	1.24	0.99–1.54	0.061	0.51	0.33	1.39
CYP1B1-AS1	1.19	0.96–1.48	0.105	0.51	0.37	1.35
LINC01936	1.26	0.91–1.74	0.163	0.51	0.43	1.39
LINC00707	1.20	0.92–1.57	0.170	0.51	0.43	1.53
AD000090.1	0.89	0.74–1.07	0.202	0.51	0.48	0.88
FP671120.6	1.11	0.94–1.32	0.219	0.51	0.50	0.96
LINC01711	1.18	0.90–1.53	0.230	0.51	0.51	1.27
AC083864.5	0.89	0.72–1.09	0.256	0.51	0.54	0.87
DNM3OS	1.09	0.92–1.29	0.323	0.53	0.59	1.18
LYPLAL1-AS1	1.11	0.90–1.36	0.333	0.53	0.60	1.25
AC046143.1	1.15	0.76–1.75	0.500	0.73	0.72	1.79
LINC01445	0.95	0.78–1.16	0.632	0.84	0.81	1.05
H19	1.02	0.92–1.14	0.684	0.84	0.84	1.04
GRASLND	0.96	0.74–1.25	0.784	0.90	0.89	1.23
AL606500.1	1.02	0.84–1.23	0.861	0.92	0.93	0.99
LINC01605	0.99	0.77–1.27	0.954	0.95	0.98	1.17

**Table 4:** Screen-nominated candidates: external validation on a comparable scale (HR per standard deviation of within-cohort expression). The pooled estimate excludes the discovery cohort.

lncRNA	TCGA Discovery, PFI	TCGA Discovery, OS	CGGA_693, OS	CGGA_325, OS	CPTAC-3, OS	Pooled External (Discovery Excluded)
LINC00707	1.27 (0.90–1.80)	1.72 (1.24–2.40)	0.93 (0.73–1.19)	1.17 (0.79–1.75)	1.15 (0.99–1.34)	1.09 (0.97–1.24), k = 3
ZFPM2-AS1	1.35 (0.99–1.84)	1.59 (1.20–2.12)	not annotated	not annotated	1.12 (0.97–1.30)	1.12 (0.97–1.30), k = 1
MROCKI	0.58 (0.41–0.82)	0.44 (0.30–0.63)	not annotated	not annotated	0.93 (0.79–1.10)	0.93 (0.79–1.10), k = 1
H19	1.07 (0.78–1.47)	1.11 (0.79–1.55)	1.14 (0.89–1.46)	0.95 (0.68–1.33)	1.14 (0.97–1.34)	1.11 (0.98–1.26), k = 3

**Table 5:** Data sources, versions and access dates.

Resource	Version/Release	Use	Accessed
TCGA-GBM RNA-seq (GDC, STAR-Counts)	GENCODE v36, GRCh38	Discovery expression	11 March 2026
cBioPortal molecular annotations	current at access	IDH, MGMT status, subtype	11 March 2026
TCGA-CDR [17]	Supplemental Table S1	PFI and OS endpoints	3 August 2026
GDC clinical/follow-up API	Data Release 45.0	Endpoint audit, treatment timing, untreated-group assessment	3 August 2026
CGGA mRNAseq_693/_325 [18]	20200506 RSEM-genes	Held-out external validation	11 March 2026
CPTAC-3 GBM RNA-seq (GDC)	STAR-Counts, GENCODE v36	Joint discovery and replication	3 August 2026
CPTAC GBM proteome/miRNA (LinkedOmics) [24]	current at access	Protein-level context	23 April 2026
GTEx	v8 median TPM	Normal-tissue context	23 April 2026
ENCORI/starBase [25]	current at access	miRNA–target interactions	23 April 2026
GDSC2 [26]	current at access	Cell-line context	23 April 2026
Ensembl REST (cross-references)	release 113	Verified alias search	3 August 2026

## Data Availability

All analysis code, the clinical-derivation rules, the sample identifiers of every cohort and all model output tables are deposited in a public GitHub repository (https://github.com/jaeilhan-sketch/lncRNA-GBM-TMZ-code, accessed on 11 March 2026), including the scripts that produced every figure and table in this manuscript. The TCGA-GBM RNA-seq and miRNA-seq data are available through the GDC (https://portal.gdc.cancer.gov/, project TCGA-GBM; accessed on 11 March 2026 for RNA-seq and clinical data, 23 April 2026 for miRNA-seq and 3 August 2026 for the clinical follow-up re-query); standardized endpoints are from the TCGA-CDR [17] (accessed 3 August 2026); CPTAC-3 GBM RNA-seq is available through the GDC (project CPTAC-3; accessed 3 August 2026); CGGA data are at http://www.cgga.org.cn (accessed 11 March 2026); ENCORI/starBase data at https://rnasysu.com/encori/ (accessed 23 April 2026); GTEx v8 at https://gtexportal.org/ (accessed 23 April 2026); CPTAC proteogenomic data at https://www.linkedomics.org/data_download/CPTAC-GBM/ (accessed 23 April 2026); GDSC2 at https://www.cancerrxgene.org/ (accessed 23 April 2026).

## Supplementary Materials

The following supporting information can be downloaded at: https://www.mdpi.com/article/10.3390/ncrna12050036/s1, Table S1: Transcriptome-wide MGMT-adjusted Cox regression of all 3785 expressed lncRNAs on (a) the progression-free interval and (b) overall survival; Table S2: (a) Covariate ladder isolating the contribution of the MGMT term and (b) bevacizumab sensitivity analysis and proportional-hazards checks; Table S3: Genome-wide MGMT-adjusted differential expression between the six-month PFI groups; Table S4: (a) Expression × MGMT interaction tests with stratum-specific hazard ratios and (b) candidate expression by MGMT methylation status; Table S5: Cellular composition: (a) marker sets used for the compartment scores, (b) correlations between candidate expression and compartment scores, (c) Cox models before and after composition adjustment and (d) composition-adjusted transcriptome-wide Cox scan; Table S6: External validation: (a) verified alias search against the CGGA annotation, (b) hazard ratios per standard deviation of within-cohort expression in TCGA and CGGA, (c) CPTAC-3 age-adjusted Cox regression on overall survival and (d) discovery-excluded pooled external estimates; Table S7: (a) Permutation-matched null for ceRNA-pattern triplet counts and (b) stoichiometry of lncRNAs, implicated miRNAs and competing target-site pools; Table S8: Eighteen widely cited glioma lncRNAs tested on the progression-free interval under MGMT adjustment; Table S9: Endpoint audit for each endpoint and cohort; Table S10: The 16 transcripts nominated by the unadjusted screen, re-tested on the progression-free interval; Table S11: Bootstrap optimism correction of the C-index, with and without repeating candidate selection inside the resample; Table S12: Joint TCGA + CPTAC-3 transcriptome-wide meta-analysis on overall survival; Table S13: DIO3OS and C9orf139 stress-tested within TCGA against MGMT status, cellular composition and endpoint; Table S14: DIO3OS and C9orf139 in the held-out CGGA cohorts; Table S15: DLK1-DIO3 imprinted locus, gene by gene, in both cohorts; Table S16: Bootstrap-corrected ΔC-index for DIO3OS and C9orf139 over the MGMT + clinical baseline; Table S17: TCGA-GBM treatment-group assessment; Table S18: Numbers at risk and cumulative events for the median-split survival curves of Figure 5. Full-length versions of Tables S1, S3, S5d and S12 and the per-sample composition scores of Table S5a are provided as tab-separated source-data files.
