# Supplementary material for: A Transcriptome-Wide Analysis Nominates DIO3OS and C9orf139 as Survival-Associated Long Non-Coding RNAs in Glioblastoma
Source: Noncoding RNA. 2026 Sep 7;12(5):36. doi: 10.3390/ncrna12050036 (PMC13600001; doi:10.3390/ncrna12050036)
Supplement: Supplementary file 1 [file ncrna-12-00036-s001.zip › Supplementary_Tables.pdf]

Supplementary Tables

Where a table is truncated for readability, the complete table is provided as a tab-separated file in the accompanying supplementary archive.

Table S1a.

Transcriptome-wide MGMT-adjusted Cox regression on progression-free interval, all 3,785 expressed lncRNAs (n = 69, 51 events). No transcript reaches FDR < 0.05.

Showing the first 40 of 3785 rows; full table: genomewide_cox_PFI_mgmt_adjusted.tsv

gene_id	gene_name	HR	lower	upper	p	fdr_transcriptomewide	endpoint
ENSG00000286342.1	AC073210.3	2.632	1.595	4.345	1.53e-04	0.3303	PFI (primary)
ENSG00000271780.1	AL118558.3	3.038	1.667	5.538	2.87e-04	0.3303	PFI (primary)
ENSG00000276846.1	AC016590.3	0.5133	0.3507	0.7513	6.01e-04	0.3303	PFI (primary)
ENSG00000273387.1	AC005005.3	3.057	1.602	5.832	6.97e-04	0.3303	PFI (primary)
ENSG00000275055.1	AC011468.5	0.5016	0.335	0.7508	8.02e-04	0.3303	PFI (primary)
ENSG00000269956.2	MKNK1-AS1	1.941	1.308	2.879	9.90e-04	0.3303	PFI (primary)
ENSG00000243107.1	AC000120.1	1.461	1.163	1.836	0.001123	0.3303	PFI (primary)
ENSG00000224376.1	AC017104.1	0.5354	0.3672	0.7807	0.001167	0.3303	PFI (primary)
ENSG00000261824.7	LINC00662	0.4099	0.2368	0.7095	0.001444	0.3303	PFI (primary)
ENSG00000253559.1	OSGEPL1-AS1	0.2853	0.1306	0.623	0.001649	0.3303	PFI (primary)
ENSG00000259863.1	SH3RF3-AS1	1.91	1.274	2.864	0.001729	0.3303	PFI (primary)
ENSG00000227502.3	MROCKI	0.6659	0.5159	0.8595	0.001792	0.3303	PFI (primary)
ENSG00000232907.8	DLGAP4-AS1	1.751	1.224	2.506	0.002159	0.3303	PFI (primary)
ENSG00000272750.1	AL592148.3	0.5241	0.3442	0.7983	0.002615	0.3303	PFI (primary)
ENSG00000226380.9	AC016831.1	1.333	1.105	1.608	0.002647	0.3303	PFI (primary)
ENSG00000254676.1	AP000873.4	1.902	1.25	2.893	0.002687	0.3303	PFI (primary)
ENSG00000259212.1	AC103739.1	1.552	1.165	2.068	0.002696	0.3303	PFI (primary)
ENSG00000272459.1	AC139795.3	0.483	0.2983	0.782	0.003073	0.3303	PFI (primary)
ENSG00000266921.2	AC006213.1	0.3627	0.1852	0.7103	0.0031	0.3303	PFI (primary)
ENSG00000253738.2	OTUD6B-AS1	0.5209	0.3362	0.807	0.003502	0.3303	PFI (primary)
ENSG00000248019.3	FAM13A-AS1	1.616	1.166	2.239	0.003916	0.3303	PFI (primary)
ENSG00000270659.1	AC079610.1	0.6354	0.4657	0.8669	0.004226	0.3303	PFI (primary)
ENSG00000257953.1	AC083805.2	1.517	1.139	2.021	0.004396	0.3303	PFI (primary)
ENSG00000253837.1	AC090197.1	1.74	1.189	2.548	0.004406	0.3303	PFI (primary)
ENSG00000232118.2	BACH1-AS1	1.654	1.168	2.342	0.004556	0.3303	PFI (primary)
ENSG00000287403.1	AC016245.2	1.457	1.123	1.89	0.004558	0.3303	PFI (primary)
ENSG00000261766.1	AC133550.3	1.928	1.222	3.041	0.004756	0.3303	PFI (primary)
ENSG00000244041.7	LINC01011	0.5621	0.3767	0.8387	0.004782	0.3303	PFI (primary)
ENSG00000235910.2	APOA1-AS	1.654	1.163	2.352	0.005092	0.3303	PFI (primary)
ENSG00000245651.3	AC083805.1	1.438	1.115	1.856	0.005172	0.3303	PFI (primary)
ENSG00000237390.1	AL139130.1	1.333	1.089	1.631	0.005265	0.3303	PFI (primary)
ENSG00000270426.1	AC099343.2	2.44	1.301	4.575	0.005421	0.3303	PFI (primary)
ENSG00000273691.1	AC087284.1	1.43	1.111	1.842	0.005505	0.3303	PFI (primary)
ENSG00000232082.1	RPS6KA2-IT1	1.589	1.145	2.206	0.005623	0.3303	PFI (primary)
ENSG00000285774.3	AL133444.1	1.365	1.095	1.702	0.005689	0.3303	PFI (primary)
ENSG00000254419.1	AL139349.1	1.734	1.174	2.562	0.005699	0.3303	PFI (primary)
ENSG00000254034.2	INTS9-AS1	1.405	1.104	1.789	0.00575	0.3303	PFI (primary)
ENSG00000204929.13	AC007389.1	1.504	1.125	2.01	0.005855	0.3303	PFI (primary)
ENSG00000213600.3	U73169.1	1.405	1.103	1.791	0.00595	0.3303	PFI (primary)

ENSG00000277806.1	AC006213.4	0.6869	0.5249	0.8988	0.006196	0.3303	PFI (primary)

Table S1b.

Transcriptome-wide MGMT-adjusted Cox regression on overall survival, all 3,785 expressed lncRNAs (n = 69, 50 deaths).

Showing the first 40 of 3785 rows; full table: genomewide_cox_OS_mgmt_adjusted.tsv

gene_id	gene_name	HR	lower	upper	p	fdr_transcriptomewide	endpoint
ENSG00000227502.3	MROCKI	0.5398	0.4095	0.7117	1.24e-05	0.04682	OS
ENSG00000277151.1	AL138820.1	2.236	1.452	3.444	2.58e-04	0.2711	OS
ENSG00000273387.1	AC005005.3	3.183	1.686	6.011	3.57e-04	0.2711	OS
ENSG00000270426.1	AC099343.2	3.783	1.822	7.855	3.57e-04	0.2711	OS
ENSG00000275494.1	AC133552.5	0.4511	0.2894	0.7031	4.40e-04	0.2711	OS
ENSG00000275557.1	AC242842.1	0.497	0.3353	0.7365	4.94e-04	0.2711	OS
ENSG00000284747.1	AL034417.4	2.095	1.378	3.186	5.45e-04	0.2711	OS
ENSG00000265625.1	AC104564.5	1.625	1.233	2.141	5.73e-04	0.2711	OS
ENSG00000276136.1	AC016957.2	2.783	1.545	5.012	6.49e-04	0.2729	OS
ENSG00000230490.3	AL139383.1	1.71	1.251	2.337	7.75e-04	0.2758	OS
ENSG00000278492.1	AC006213.5	0.3982	0.2309	0.6868	9.30e-04	0.2758	OS
ENSG00000234263.2	MAP3K5-AS1	1.963	1.316	2.929	9.50e-04	0.2758	OS
ENSG00000272106.1	AL691432.2	0.5192	0.3507	0.7688	0.001063	0.2758	OS
ENSG00000272630.1	AL731563.3	3.209	1.596	6.453	0.001072	0.2758	OS
ENSG00000269834.6	ZNF528-AS1	0.4905	0.3196	0.7529	0.001119	0.2758	OS
ENSG00000286342.1	AC073210.3	2.166	1.354	3.466	0.001274	0.2758	OS
ENSG00000238266.2	LINC00707	1.517	1.176	1.957	0.001329	0.2758	OS
ENSG00000286710.1	AC188617.1	0.6878	0.5473	0.8645	0.001333	0.2758	OS
ENSG00000251003.9	ZFPM2-AS1	1.393	1.137	1.706	0.001385	0.2758	OS
ENSG00000248668.3	OXCT1-AS1	0.591	0.4258	0.8204	0.001671	0.2911	OS
ENSG00000261105.6	LMO7-AS1	2.196	1.342	3.594	0.001744	0.2911	OS
ENSG00000271833.1	AL445222.1	2.784	1.466	5.29	0.001763	0.2911	OS
ENSG00000267454.6	ZNF582-AS1	0.5905	0.4243	0.8219	0.001789	0.2911	OS
ENSG00000181908.5	LINC02724	0.5851	0.4176	0.8199	0.001846	0.2911	OS
ENSG00000259943.1	AL050341.2	0.5153	0.3377	0.7861	0.002096	0.3173	OS
ENSG00000180539.8	C9orf139	2.065	1.293	3.297	0.002407	0.3503	OS
ENSG00000222012.1	AC005481.1	1.446	1.138	1.837	0.002518	0.353	OS
ENSG00000243224.1	AC006252.1	1.864	1.237	2.81	0.002934	0.3966	OS
ENSG00000229334.1	AC046143.1	1.749	1.206	2.535	0.00318	0.4151	OS
ENSG00000227640.3	SOX21-AS1	0.683	0.5291	0.8816	0.003413	0.4274	OS
ENSG00000272523.1	LINC01023	0.4952	0.3086	0.7945	0.003573	0.4274	OS
ENSG00000258584.2	FAM181A-AS1	0.7168	0.5719	0.8984	0.003855	0.4274	OS
ENSG00000197301.7	HMGA2-AS1	1.411	1.117	1.783	0.003928	0.4274	OS
ENSG00000255052.5	FAM66D	1.99	1.246	3.179	0.003992	0.4274	OS
ENSG00000228592.2	D21S2088E	0.6334	0.4635	0.8657	0.004174	0.4274	OS
ENSG00000229267.3	SNHG31	1.809	1.205	2.716	0.00425	0.4274	OS
ENSG00000234997.2	AC016745.1	1.612	1.16	2.24	0.00446	0.4274	OS
ENSG00000261997.1	AC007336.1	1.617	1.161	2.254	0.004515	0.4274	OS
ENSG00000259705.1	AC084757.3	0.5543	0.3686	0.8335	0.004579	0.4274	OS
ENSG00000287277.1	AL392086.3	1.522	1.138	2.038	0.004707	0.4274	OS

Table S2a.

Covariate ladder: identical genes in identical patients, covariates added one at a time, isolating the contribution of the MGMT term (endpoint PFI).

gene	n	events	HR_unadj	p_unadj	HR_MGMTonly	p_MGMTonly	HR_MGMT_age_sex	p_MGMT_age_sex	HR_full	p_full	pct_HR_change_from_MGMT
LINC00707	69	51	1.168	0.2342	1.205	0.1676	1.203	0.1697	1.23	0.1306	3.137
ZFPM2-AS1	69	51	1.213	0.07032	1.22	0.06587	1.236	0.06113	1.235	0.06215	0.5276
LINC01936	69	51	1.252	0.1709	1.227	0.216	1.26	0.1631	1.319	0.09137	-1.98
AC083864.5	69	51	0.8896	0.263	0.8793	0.2232	0.8862	0.2563	0.888	0.241	-1.163
H19	69	51	1.028	0.6264	1.022	0.6927	1.023	0.6841	1.037	0.5208	-0.5183
DNM3OS	69	51	1.075	0.3913	1.079	0.3535	1.089	0.3231	1.091	0.3314	0.4601
MROCKI	69	51	0.6863	0.002314	0.6911	0.003426	0.6659	0.001792	0.675	0.002014	0.698
CYP1B1-AS1	69	51	1.205	0.07833	1.189	0.1086	1.194	0.1049	1.22	0.08239	-1.32

Table S2b.

Bevacizumab sensitivity analysis and proportional-hazards checks (scaled Schoenfeld residuals).

gene	HR_noBev	HR_withBev	pct_change	zph_expr_p	zph_global_p
LINC00707	1.203	1.23	2.174	0.1624	0.06496
ZFPM2-AS1	1.236	1.235	0.1092	0.1637	0.05673
MROCKI	0.6659	0.675	1.36	0.9597	0.3205
LINC01936	1.26	1.319	4.688	0.4658	0.1398
H19	1.023	1.037	1.322	0.07511	0.03655
DNM3OS	1.089	1.091	0.1989	0.4323	0.1704
AC083864.5	0.8862	0.888	0.1983	0.06858	0.07058
CYP1B1-AS1	1.194	1.22	2.213	0.3143	0.1372

Table S3.

Genome-wide MGMT-adjusted differential expression between the six-month PFI groups (n = 61; 25 early, 36 late).

Showing the first 40 of 3785 rows; full table: genomewide_DE_PFI6mo_mgmt_adjusted.tsv

gene_id	baseMean	log2FoldChange	lfcSE	stat	pvalue	padj	gene_name
ENSG00000223812.8	226.2	-3.228	0.6463	-4.994	5.91e-07	0.001715	AC073365.1
ENSG00000244055.2	481.3	1.581	0.3375	4.685	2.80e-06	0.002714	AC007566.1
ENSG00000245651.3	48.77	1.903	0.3998	4.761	1.93e-06	0.002714	AC083805.1
ENSG00000267014.6	78.48	-2.612	0.5826	-4.483	7.37e-06	0.00535	LINC01532
ENSG00000231290.6	112.9	1.881	0.4413	4.261	2.03e-05	0.01181	APCDD1L-DT
ENSG00000231918.1	40.44	-2.114	0.5169	-4.09	4.32e-05	0.02021	AC007402.1
ENSG00000251003.9	75.91	1.791	0.441	4.062	4.87e-05	0.02021	ZFPM2-AS1
ENSG00000270039.1	156.2	1.405	0.3564	3.943	8.04e-05	0.0292	AC025165.5
ENSG00000188693.8	44.93	1.396	0.3666	3.808	1.40e-04	0.03302	CYP51A1-AS1
ENSG00000227502.3	381.4	-1.183	0.3117	-3.795	1.48e-04	0.03302	MROCKI
ENSG00000234707.3	275.9	-2.359	0.6174	-3.821	1.33e-04	0.03302	SEC61G-DT
ENSG00000238266.2	37.06	1.729	0.4509	3.834	1.26e-04	0.03302	LINC00707
ENSG00000260423.2	51.4	-1.625	0.4219	-3.852	1.17e-04	0.03302	LINC02367
ENSG00000287893.1	44.23	-1.825	0.4953	-3.686	2.28e-04	0.04735	AC083864.5
ENSG00000240086.7	44.69	-1.72	0.4823	-3.566	3.63e-04	0.07029	AC092969.1
ENSG00000250451.5	32.61	-1.932	0.5591	-3.456	5.48e-04	0.09944	HOXC-AS1

ENSG00000253553.7	67.12	-0.6514	0.1951	-3.339	8.41e-04	0.1356	AC090578.1
ENSG00000278811.4	85.51	1.467	0.4394	3.34	8.38e-04	0.1356	LINC00624
ENSG00000236861.6	24.48	0.8678	0.2707	3.206	0.001347	0.1968	BET1-AS1
ENSG00000269903.1	34.33	1.103	0.3442	3.204	0.001355	0.1968	AC025165.4
ENSG00000271780.1	89.38	0.5611	0.1771	3.168	0.001538	0.2126	AL118558.3
ENSG00000186235.11	58.72	-1.035	0.3348	-3.093	0.001981	0.2601	LINC02610
ENSG00000204380.4	43.3	-1.098	0.3572	-3.073	0.002116	0.2601	PKP4-AS1
ENSG00000204446.4	43.15	-0.6666	0.2172	-3.069	0.002149	0.2601	LINC02872
ENSG00000234377.7	102.7	-1.127	0.3808	-2.959	0.003086	0.3505	OBI1-AS1
ENSG00000274536.7	118.4	0.8254	0.2794	2.954	0.003138	0.3505	MIR223HG
ENSG00000272274.2	50.27	1.086	0.3706	2.931	0.003376	0.3631	LINC00551
ENSG00000257953.1	36.6	1.157	0.4035	2.868	0.00413	0.4283	AC083805.2
ENSG00000259225.7	24.88	0.9509	0.3348	2.84	0.004509	0.4515	LINC02345
ENSG00000238078.1	26.5	-0.9543	0.3398	-2.808	0.004986	0.467	LINC01352
ENSG00000266921.2	44.73	-0.4526	0.161	-2.811	0.004935	0.467	AC006213.1
ENSG00000287867.1	31.27	-1.597	0.5771	-2.767	0.005658	0.5135	AC007402.2
ENSG00000231817.9	75.41	1.473	0.5384	2.736	0.00621	0.5457	LINC01198
ENSG00000284747.1	25.99	0.8701	0.3191	2.727	0.006389	0.5457	AL034417.4
ENSG00000189238.6	58.03	1.278	0.4753	2.688	0.007199	0.5586	LINC00943
ENSG00000237390.1	110.4	1.251	0.4665	2.682	0.007309	0.5586	AL139130.1
ENSG00000254139.1	87.88	-1.332	0.4934	-2.7	0.006935	0.5586	AC104051.2
ENSG00000259863.1	97.57	0.6084	0.2268	2.683	0.007296	0.5586	SH3RF3-AS1
ENSG00000255495.1	34.81	0.7238	0.2715	2.666	0.007682	0.5674	AC145124.1
ENSG00000269054.2	69.6	-0.5054	0.19	-2.66	0.007816	0.5674	AC012313.6

Table S4a.

Expression x MGMT interaction tests with stratum-specific hazard ratios and event counts, in place of descriptive robustness tiers.

gene	HR_methylated	n_meth	ev_meth	HR_unmethylated	n_unmeth	ev_unmeth	interaction_HR	interaction_p	LRT_p	interaction_fdr
LINC00707	1.049	34	26	1.315	35	25	1.168	0.5646	0.5689	0.8945
ZFPM2-AS1	1.219	34	26	1.146	35	25	0.8234	0.3951	0.3907	0.8945
LINC01936	3.468	34	26	1.148	35	25	0.2922	0.01417	0.008419	0.1133
AC083864.5	0.8926	34	26	0.8348	35	25	1.035	0.872	0.8721	0.8945
H19	0.9727	34	26	1.028	35	25	1.063	0.6146	0.6148	0.8945
DNM3OS	1.014	34	26	1.184	35	25	1.048	0.7892	0.7883	0.8945
MROCKI	0.7246	34	26	0.6268	35	25	0.966	0.8945	0.8945	0.8945
CYP1B1-AS1	1.254	34	26	1.249	35	25	0.8735	0.5806	0.5789	0.8945

Table S4b.

Candidate expression by MGMT methylation status (Wilcoxon and t tests, BH-corrected).

gene	mean_methylated	mean_unmethylated	diff_VST	wilcox_p	t_p	wilcox_fdr
LINC00707	5.064	4.835	0.2292	0.2567	0.4698	0.6252
ZFPM2-AS1	5.649	5.534	0.1153	0.752	0.7367	0.9011
LINC01936	4.971	5.077	-0.1066	0.9857	0.6109	0.9857
AC083864.5	4.741	4.981	-0.2402	0.7687	0.485	0.9011
H19	7.91	9.03	-1.12	0.1235	0.1026	0.4941
DNM3OS	5.153	5.411	-0.2577	0.7885	0.4756	0.9011

MROCKI	8.377	7.821	0.5562	0.1235	0.08585	0.4941
CYP1B1-AS1	4.923	5.418	-0.4949	0.3126	0.135	0.6252

Table S5a.

Marker sets used to compute the bulk-tissue compartment scores. Scores are the mean of z-scored, variance-stabilizing-transformed expression of the markers detected in the cohort; they are marker-based compartment scores, not reference-based deconvolution or DNA-derived purity estimates. Per-sample scores are provided as TableS5a_composition_scores.tsv.

Compartment	Markers
Myeloid	PTPRC, CD68, AIF1, CSF1R, ITGAM, TYROBP, FCER1G, C1QA, C1QB, CD163, MRC1
Lymphoid	CD3D, CD3E, CD2, CD8A, IL7R, GZMA, SKAP1
Endothelial	PECAM1, VWF, CDH5, CLDN5, FLT1, ENG
Stromal	COL1A1, COL1A2, COL3A1, FN1, DCN, LUM, THY1
Neuronal	RBFOX3, SYP, SNAP25, SYT1, NEFL, GRIN1, STMN2
Oligodendroglial	MBP, PLP1, MOG, MAG, CNP, MOBP
Proliferation	MKI67, TOP2A, CCNB1, BUB1, AURKA, PLK1
Hypoxia / necrosis	VEGFA, CA9, ADM, NDRG1, SLC2A1, PGK1, LDHA

Table S5b.

Spearman correlations between candidate lncRNA expression and marker-based compartment scores.

compartment	rho	p	gene	fdr
myeloid	0.2655	0.009701	LINC00707	0.04939
lymphoid	0.06538	0.5313	LINC00707	0.7068
endothelial	-0.1593	0.1251	LINC00707	0.3184
stromal	0.05964	0.568	LINC00707	0.7068
neuronal	0.04177	0.6894	LINC00707	0.7756
oligodendro	0.0792	0.4479	LINC00707	0.6271
proliferation	0.04132	0.6925	LINC00707	0.7756
hypoxia_necrosis	-0.01486	0.8869	LINC00707	0.9372
myeloid	0.2301	0.02586	ZFPM2-AS1	0.09656
lymphoid	0.0106	0.9191	ZFPM2-AS1	0.9379
endothelial	0.2595	0.01174	ZFPM2-AS1	0.0548
stromal	0.4218	2.74e-05	ZFPM2-AS1	7.38e-04
neuronal	0.05176	0.6197	ZFPM2-AS1	0.7544
oligodendro	0.1642	0.1136	ZFPM2-AS1	0.303
proliferation	-0.08396	0.4204	ZFPM2-AS1	0.6158
hypoxia_necrosis	0.125	0.2297	ZFPM2-AS1	0.4436
myeloid	-0.1486	0.1527	MROCKI	0.3562
lymphoid	-0.09703	0.3515	MROCKI	0.579
endothelial	0.1183	0.2554	MROCKI	0.4614
stromal	-0.112	0.2819	MROCKI	0.4934
neuronal	0.002868	0.9781	MROCKI	0.9781
oligodendro	0.04182	0.6885	MROCKI	0.7756
proliferation	-0.01034	0.9211	MROCKI	0.9379
hypoxia_necrosis	-0.08667	0.4055	MROCKI	0.6137
myeloid	0.05961	0.5676	LINC01936	0.7068
lymphoid	0.06653	0.5234	LINC01936	0.7068
endothelial	0.2264	0.02843	LINC01936	0.09951
stromal	0.2553	0.01322	LINC01936	0.05696

neuronal	-0.1377	0.1855	LINC01936	0.371
oligodendro	-0.09192	0.3776	LINC01936	0.6042
proliferation	0.1648	0.1125	LINC01936	0.303
hypoxia_necrosis	0.3992	7.70e-05	LINC01936	8.63e-04
myeloid	0.02321	0.824	H19	0.8874
lymphoid	-0.03022	0.7721	H19	0.8478
endothelial	0.1191	0.2525	H19	0.4614
stromal	0.3102	0.002445	H19	0.01712
neuronal	0.08797	0.3985	H19	0.6137
oligodendro	0.138	0.1845	H19	0.371
proliferation	0.4024	6.69e-05	H19	8.63e-04
hypoxia_necrosis	0.3696	2.71e-04	H19	0.002527
myeloid	0.178	0.08619	DNM3OS	0.254
lymphoid	0.1417	0.1728	DNM3OS	0.371
endothelial	0.1525	0.142	DNM3OS	0.3458
stromal	0.4939	6.09e-07	DNM3OS	3.41e-05
neuronal	-0.2161	0.03661	DNM3OS	0.1206
oligodendro	-0.1062	0.3077	DNM3OS	0.5222
proliferation	0.06185	0.5531	DNM3OS	0.7068
hypoxia_necrosis	-0.04558	0.6621	DNM3OS	0.7756
myeloid	-0.3358	9.35e-04	AC083864.5	0.007483
lymphoid	-0.2781	0.006648	AC083864.5	0.03723
endothelial	-0.1842	0.07559	AC083864.5	0.2352
stromal	-0.4106	3.95e-05	AC083864.5	7.38e-04
neuronal	0.2315	0.02479	AC083864.5	0.09656
oligodendro	0.2798	0.006305	AC083864.5	0.03723
proliferation	0.1386	0.1829	AC083864.5	0.371
hypoxia_necrosis	-0.08256	0.4289	AC083864.5	0.6158

Table S5c.

Cox models before and after adjustment for cellular composition, PFI and OS.

endpoint	gene	HR_base	p_base	HR_composition_adj	lo	hi	p_composition_adj
PFI	LINC00707	1.273	0.1697	1.272	0.8443	1.917	0.2496
PFI	ZFPM2-AS1	1.348	0.06113	1.526	1.055	2.207	0.02481
PFI	MROCKI	0.5781	0.001792	0.5483	0.3791	0.7929	0.00141
PFI	LINC01936	1.221	0.1631	1.273	0.9164	1.769	0.1499
PFI	H19	1.068	0.6841	1.118	0.7337	1.703	0.6042
PFI	DNM3OS	1.136	0.3231	1.677	0.9687	2.904	0.06484
PFI	AC083864.5	0.8427	0.2563	0.8058	0.5617	1.156	0.2409
OS	LINC00707	1.723	0.001329	1.9	1.304	2.767	8.25e-04
OS	ZFPM2-AS1	1.594	0.001385	1.918	1.37	2.687	1.51e-04
OS	MROCKI	0.4356	1.24e-05	0.3924	0.2625	0.5866	5.10e-06
OS	LINC01936	1.32	0.06003	1.346	0.9755	1.858	0.07043
OS	H19	1.105	0.56	1.22	0.784	1.898	0.3784
OS	DNM3OS	1.279	0.04274	2.669	1.481	4.811	0.001088
OS	AC083864.5	0.8192	0.2179	0.7206	0.4903	1.059	0.09544

Table S5d.

Transcriptome-wide Cox scan repeated with composition adjustment (top rows; full table in the accompanying file).

Showing the first 40 of 7570 rows; full table: genomewide_cox_composition_adjusted.tsv

gene_id	gene_name	HR	p	fdr	endpoint
ENSG00000286342.1	AC073210.3	2.828	7.41e-05	0.2081	PFI
ENSG00000276846.1	AC016590.3	0.4216	1.77e-04	0.2081	PFI
ENSG00000271780.1	AL118558.3	3.946	2.31e-04	0.2081	PFI
ENSG00000273387.1	AC005005.3	3.661	3.35e-04	0.2081	PFI
ENSG00000224376.1	AC017104.1	0.4637	3.56e-04	0.2081	PFI
ENSG00000274536.7	MIR223HG	2.953	3.68e-04	0.2081	PFI
ENSG00000259863.1	SH3RF3-AS1	2.235	3.85e-04	0.2081	PFI
ENSG00000269956.2	MKNK1-AS1	2.236	4.67e-04	0.2207	PFI
ENSG00000243107.1	AC000120.1	1.575	5.61e-04	0.2207	PFI
ENSG00000275055.1	AC011468.5	0.4158	6.35e-04	0.2207	PFI
ENSG00000269903.1	AC025165.4	1.887	7.36e-04	0.2207	PFI
ENSG00000254676.1	AP000873.4	2.115	7.75e-04	0.2207	PFI
ENSG00000257953.1	AC083805.2	1.677	8.48e-04	0.2207	PFI
ENSG00000244041.7	LINC01011	0.4819	9.57e-04	0.2207	PFI
ENSG00000254419.1	AL139349.1	2.207	9.94e-04	0.2207	PFI
ENSG00000259212.1	AC103739.1	1.785	0.001004	0.2207	PFI
ENSG00000259762.1	AC021739.4	1.88	0.001263	0.2207	PFI
ENSG00000272323.1	AC026801.2	0.5414	0.001278	0.2207	PFI
ENSG00000259933.6	AC009065.2	1.856	0.00129	0.2207	PFI
ENSG00000227502.3	MROCKI	0.6402	0.00141	0.2207	PFI
ENSG00000251034.2	AC087854.1	1.984	0.001444	0.2207	PFI
ENSG00000235910.2	APOA1-AS	1.875	0.00154	0.2207	PFI
ENSG00000226067.7	LINC00623	0.6259	0.001585	0.2207	PFI
ENSG00000272459.1	AC139795.3	0.4315	0.001598	0.2207	PFI
ENSG00000228484.3	AL137025.1	0.3938	0.001655	0.2207	PFI
ENSG00000283031.2	AC009242.1	1.558	0.001721	0.2207	PFI
ENSG00000253559.1	OSGEPL1-AS1	0.2289	0.001745	0.2207	PFI
ENSG00000259163.2	AL096869.2	1.829	0.001751	0.2207	PFI
ENSG00000275557.1	AC242842.1	0.4885	0.001766	0.2207	PFI
ENSG00000266921.2	AC006213.1	0.3436	0.002035	0.2207	PFI
ENSG00000186235.11	LINC02610	0.5363	0.002039	0.2207	PFI
ENSG00000257703.6	AC068643.1	0.5984	0.0022	0.2207	PFI
ENSG00000261766.1	AC133550.3	2.229	0.002206	0.2207	PFI
ENSG00000261824.7	LINC00662	0.3626	0.00227	0.2207	PFI
ENSG00000232082.1	RPS6KA2-IT1	1.718	0.002357	0.2207	PFI
ENSG00000270060.1	AC090589.3	2.187	0.002398	0.2207	PFI
ENSG00000259081.1	AF111169.3	2.07	0.002428	0.2207	PFI
ENSG00000232907.8	DLGAP4-AS1	1.766	0.002428	0.2207	PFI
ENSG00000287104.1	AC097382.3	0.4541	0.002442	0.2207	PFI
ENSG00000245651.3	AC083805.1	1.547	0.002544	0.2207	PFI

Table S6a.

Verified Ensembl cross-reference alias search against the CGGA RSEM-genes annotation (1-169 identifiers searched per transcript).

gene	ensembl_gene_id	cohort	n_verified_aliases	verified_aliases	found_in_cgga_as	available
LINC00707	ENSG00000238266	CGGA_693	1	LINC00707	LINC00707	True
LINC00707	ENSG00000238266	CGGA_325	1	LINC00707	LINC00707	True
ZFPM2-AS1	ENSG00000251003	CGGA_693	32	50698|HGNC:50698|NR_125796|NR_125796.1|NR_125797|NR_125797.1|SCAT3|URS00008B393A|URS00008B40B6|URS00008B55BA|URS00008B86EF|URS00008BBCA4|URS0000A80630|URS0000DBB575|URS0000EEC8D3|URS0002A18320|URS0002A25265|URS0002A342F3|URS0002A42034|ZFPM2-AS1|ZFPM2-AS1-201|ZFPM2-AS1-202|ZFPM2-AS1-203|ZFPM2-AS1-204|ZFPM2-AS1-205|ZFPM2-AS1-206|ZFPM2-AS1-207|ZFPM2-AS1-208|ZFPM2-AS1-209|ZFPM2-AS1-210|ZFPM2-AS1-211|ZFPM2-AS1-212		False
ZFPM2-AS1	ENSG00000251003	CGGA_325	32	50698|HGNC:50698|NR_125796|NR_125796.1|NR_125797|NR_125797.1|SCAT3|URS00008B393A|URS00008B40B6|URS00008B55BA|URS00008B86EF|URS00008BBCA4|URS0000A80630|URS0000DBB575|URS0000EEC8D3|URS0002A18320|URS0002A25265|URS0002A342F3|URS0002A42034|ZFPM2-AS1|ZFPM2-AS1-201|ZFPM2-AS1-202|ZFPM2-AS1-203|ZFPM2-AS1-204|ZFPM2-AS1-205|ZFPM2-AS1-206|ZFPM2-AS1-207|ZFPM2-AS1-208|ZFPM2-AS1-209|ZFPM2-AS1-210|ZFPM2-AS1-211|ZFPM2-AS1-212		False
MROCKI	ENSG00000227502	CGGA_693	33	285758|50323|HGNC:50323|LINC01268|LOC285758|MROCKI|MROCKI-201|MROCKI-202|MROCKI-203|MROCKI-204|MROCKI-205|MROCKI-206|MROCKI-207|MROCKI-208|MROCKI-209|MROCKI-210|MROCKI-211|MROCKI-212|NR_038863|NR_038863.2|ROCKI|URS0000EF2C42|URS00022B8FA2|URS0002A1D112|URS0002A1D76E|URS0002A267E5|URS0002A2988C|URS0002A3C8C1|URS0002A3E87B|URS0002A486DD|URS0002A5169D|URS0002A5E00D|URS0002A69BAF		False
MROCKI	ENSG00000227502	CGGA_325	33	285758|50323|HGNC:50323|LINC01268|LOC285758|MROCKI|MROCKI-201|MROCKI-202|MROCKI-203|MROCKI-204|MROCKI-205|MROCKI-206|MROCKI-207|MROCKI-208|MROCKI-209|MROCKI-210|MROCKI-211|MROCKI-212|NR_038863|NR_038863.2|ROCKI|URS0000EF2C42|URS00022B8FA2|URS0002A1D112|URS0002A1D76E|URS0002A267E5|URS0002A2988C|URS0002A3C8C1|URS0002A3E87B|URS0002A486DD|URS0002A5169D|URS0002A5E00D|URS0002A69BAF		False
LINC01936	ENSG00000235997	CGGA_693	15	52759|HGNC:52759|LINC01936|LINC01936-201|LINC01936-202|LINC01936-203|LINC01936-204|LINC01936-205|NR_122048|NR_122048.1|URS000058EA2B|URS0000EF37FB|URS0000EF3D5E|URS0002A3F486|URS0002A4F33C		False
LINC01936	ENSG00000235997	CGGA_325	15	52759|HGNC:52759|LINC01936|LINC01936-201|LINC01936-202|LINC01936-203|LINC01936-204|LINC01936-205|NR_122048|NR_122048.1|URS000058EA2B|URS00		False

				00EF37FB|URS0000EF3D5E|URS0002A3F486|URS0002A4F33C		
AC083864.5	ENSG00000287893	CGGA_693	17	124901616|AC083864.5|LOC124901616|URS0000EF378B|URS0002A1D765|URS0002A2A180|URS0002A2C627|URS0002A3BDD8|URS0002A4CC37|URS0002A53492|URS0002A59316|URS0002A5B53C|URS0002A5E49E|XR_001745010|XR_001745010.1|XR_007060288|XR_007060288.1		False
AC083864.5	ENSG00000287893	CGGA_325	17	124901616|AC083864.5|LOC124901616|URS0000EF378B|URS0002A1D765|URS0002A2A180|URS0002A2C627|URS0002A3BDD8|URS0002A4CC37|URS0002A53492|URS0002A59316|URS0002A5B53C|URS0002A5E49E|XR_001745010|XR_001745010.1|XR_007060288|XR_007060288.1		False
H19	ENSG00000130600	CGGA_693	123	103280|130650|194070|283120|4713|ASM|ASM1|BECKWITH-WIEDEMANN SYNDROME; BWS [#130650]|D11S813E|GMRSP|H19|H19, IMPRINTED MATERNALLY EXPRESSED NONCODING TRANSCRIPT; H19 [*103280]|H19-201|H19-202|H19-203|H19-204|H19-205|H19-206|H19-207|H19-208|H19-209|H19-210|H19-211|H19-212|H19-213|H19-214|H19-215|H19-216|H19-217|H19-218|H19-219|H19-220|H19-221|H19-222|H19-223|H19-224|H19-225|H19-226|H19-227|H19-228|H19-229|H19-230|H19-231|H19-232|H19-233|H19-234|H19-235|H19-236|H19-237|H19-238|H19-239|H19-240|H19-241|H19-242|H19-243|H19-244|H19-245|H19-246|HGNC:4713|LINC00008|LRG_1030|LRG_1030t1|MIR675HG|NCRNA00008|NR_002196|NR_002196.3|NR_131223|NR_131223.2|NR_131224|NR_131224.2|NR_185828|NR_185828.1|NR_185829|NR_185829.1|NR_185830|NR_185830.1|NR_185831|NR_185831.1|R-HSA-212436|R-HSA-73857|R-HSA-74160|R-HSA-8878171|R-HSA-8934593|URS0000D6509C|URS00022B637B|URS00022B6714|URS00022B7AAD|URS00025E79E8|URS00026A1E43|URS00027E822F|URS00027ED209|URS00027F0DDF|URS00027F21B1|URS00027F27DF|URS00027F3093|URS00027F6F9C|URS00027F98F6|URS00027FA8E4|URS00027FAEDE|URS00027FCBE3|URS00027FCD42|URS00027FD180|URS00027FDD9F|URS0002A15CD2|URS0002A22767|URS0002A23C76|URS0002A2A3F7|URS0002A2EFB4|URS0002A334DA|URS0002A3733B|URS0002A466BF|URS0002A4BF17|URS0002A502B3|URS0002A54246|URS0002A54411|URS0002A5801F|URS0002A59BAE|URS0002A5CFF6|URS0002A5EF8D|URS0002A645F9|URS0002A68EDC|URS0002A6E3AF|WILMS TUMOR 1; WT1 [#194070]	H19	True
H19	ENSG00000130600	CGGA_325	123	103280|130650|194070|283120|4713|ASM|ASM1|BECKWITH-WIEDEMANN SYNDROME; BWS [#130650]|D11S813E|GMRSP|H19|H19, IMPRINTED MATERNALLY EXPRESSED NONCODING TRANSCRIPT; H19 [*103280]|H19-201|H19-202|H19-203|H19-204|H19-205|H19-206|H19-207|H19-208|H19-209|H19-210|H19-211|H19-212|H19-213|H19-214|H19-215|H19-216|H19-217|H19-218|H19-219|H19-220|H19-221|H19-222|H19-223|H19-224|H19-225|H19-226|H19-	H19	True

				227|H19-228|H19-229|H19-230|H19-231|H19-232|H19-233|H19-234|H19-235|H19-236|H19-237|H19-238|H19-239|H19-240|H19-241|H19-242|H19-243|H19-244|H19-245|H19-246|HGNC:4713|LINC00008|LRG_1030|LRG_1030t1|MIR675HG|NCRNA00008|NR_002196|NR_002196.3|NR_131223|NR_131223.2|NR_131224|NR_131224.2|NR_185828|NR_185828.1|NR_185829|NR_185829.1|NR_185830|NR_185830.1|NR_185831|NR_185831.1|R-HSA-212436|R-HSA-73857|R-HSA-74160|R-HSA-8878171|R-HSA-8934593|URS0000D6509C|URS00022B637B|URS00022B6714|URS00022B7AAD|URS00025E79E8|URS00026A1E43|URS00027E822F|URS00027ED209|URS00027F0DDF|URS00027F21B1|URS00027F27DF|URS00027F3093|URS00027F6F9C|URS00027F98F6|URS00027FA8E4|URS00027FAEDE|URS00027FCBE3|URS00027FCD42|URS00027FD180|URS00027FDD9F|URS0002A15CD2|URS0002A22767|URS0002A23C76|URS0002A2A3F7|URS0002A2EFB4|URS0002A334DA|URS0002A3733B|URS0002A466BF|URS0002A4BF17|URS0002A502B3|URS0002A54246|URS0002A54411|URS0002A5801F|URS0002A59BAE|URS0002A5CFF6|URS0002A5EF8D|URS0002A645F9|URS0002A68EDC|URS0002A6E3AF|WILMS TUMOR 1; WT1 [#194070]		
DNM3OS	ENSG00000230630	CGGA_693	25	41228|DNM3-AS1|DNM3OS|DNM3OS-201|DNM3OS-202|DNM3OS-203|DNM3OS-204|DNM3OS-205|DNM3OS-206|DNM3OS-207|GO:0016442|GO:0035195|HGNC:41228|MIR199A2HG|NR_038397|NR_038397.2|NR_103486|NR_103486.1|URS0000DB7639|URS0000DB80EB|URS0000DB8675|URS0000DBB0F3|URS0002A410B1|URS0002A5BFBE|URS0002A69264		False
DNM3OS	ENSG00000230630	CGGA_325	25	41228|DNM3-AS1|DNM3OS|DNM3OS-201|DNM3OS-202|DNM3OS-203|DNM3OS-204|DNM3OS-205|DNM3OS-206|DNM3OS-207|GO:0016442|GO:0035195|HGNC:41228|MIR199A2HG|NR_038397|NR_038397.2|NR_103486|NR_103486.1|URS0000DB7639|URS0000DB80EB|URS0000DB8675|URS0000DBB0F3|URS0002A410B1|URS0002A5BFBE|URS0002A69264	DNM3OS	True
CYP1B1-AS1	ENSG00000232973	CGGA_693	169	285154|28543|C2ORF58|CYP1B1-AS1|CYP1B1-AS1-201|CYP1B1-AS1-202|CYP1B1-AS1-203|CYP1B1-AS1-204|CYP1B1-AS1-205|CYP1B1-AS1-206|CYP1B1-AS1-207|CYP1B1-AS1-208|CYP1B1-AS1-209|CYP1B1-AS1-210|CYP1B1-AS1-211|CYP1B1-AS1-212|CYP1B1-AS1-213|CYP1B1-AS1-214|CYP1B1-AS1-215|CYP1B1-AS1-216|CYP1B1-AS1-217|CYP1B1-AS1-218|CYP1B1-AS1-219|CYP1B1-AS1-220|CYP1B1-AS1-221|CYP1B1-AS1-222|CYP1B1-AS1-223|CYP1B1-AS1-224|CYP1B1-AS1-225|CYP1B1-AS1-226|CYP1B1-AS1-227|CYP1B1-AS1-228|CYP1B1-AS1-229|CYP1B1-AS1-230|CYP1B1-AS1-231|CYP1B1-AS1-232|CYP1B1-AS1-233|CYP1B1-AS1-234|CYP1B1-AS1-235|CYP1B1-AS1-236|CYP1B1-AS1-237|CYP1B1-AS1-238|CYP1B1-AS1-239|CYP1B1-AS1-240|CYP1B1-AS1-241|CYP1B1-AS1-242|CYP1B1-AS1-243|CYP1B1-AS1-244|CYP1B1-AS1-245|CYP1B1-AS1-	CYP1B1-AS1	True

				246|CYP1B1-AS1-247|CYP1B1-AS1-248|CYP1B1-AS1-249|CYP1B1-AS1-250|CYP1B1-AS1-251|CYP1B1-AS1-252|CYP1B1-AS1-253|CYP1B1-AS1-254|CYP1B1-AS1-255|CYP1B1-AS1-256|CYP1B1-AS1-257|CYP1B1-AS1-258|CYP1B1-AS1-259|CYP1B1-AS1-260|CYP1B1-AS1-261|CYP1B1-AS1-262|CYP1B1-AS1-263|CYP1B1-AS1-264|CYP1B1-AS1-265|CYP1B1-AS1-266|CYP1B1-AS1-267|CYP1B1-AS1-268|CYP1B1-AS1-269|CYP1B1-AS1-270|CYP1B1-AS1-271|CYP1B1-AS1-272|CYP1B1-AS1-273|CYP1B1-AS1-274|CYP1B1-AS1-275|CYP1B1-AS1-276|CYP1B1-AS1-277|CYP1B1-AS1-278|GO:0000244|GO:0000353|GO:0005688|GO:0030621|GO:0046540|HGNC:28543|MGC34824|NR_027252|NR_027252.1|URS000046253D|URS00004723A8|URS00004C21D3|URS00004C2A84|URS00004FF70A|URS000076C4F9|URS000078116B|URS00008116DF|URS00008117C8|URS0000811815|URS0000811920|URS0000811A98|URS0000811C13|URS0000811D03|URS0000811D18|URS0000811D54|URS0000811DBA|URS0000811DE5|URS0000811F29|URS0000812033|URS0000DB8835|URS0000EEF01A|URS0000EF552E|URS0000EF6824|URS00022B3FF8|URS00025E62BF|URS00025F35FB|URS0002A17B38|URS0002A19198|URS0002A1BF8E|URS0002A2537B|URS0002A27E89|URS0002A29523|URS0002A297A2|URS0002A29854|URS0002A2B765|URS0002A2D853|URS0002A2F09D|URS0002A30A26|URS0002A30ADA|URS0002A30AEE|URS0002A30F4D|URS0002A3416F|URS0002A34A81|URS0002A34B20|URS0002A36BE5|URS0002A39C62|URS0002A3D24A|URS0002A3D996|URS0002A3E21F|URS0002A41557|URS0002A4367E|URS0002A44257|URS0002A448A7|URS0002A47699|URS0002A495A4|URS0002A49981|URS0002A49E4B|URS0002A4D694|URS0002A4FB2B|URS0002A50437|URS0002A568D2|URS0002A5ADDB|URS0002A5B64D|URS0002A5C612|URS0002A5DC06|URS0002A5DD71|URS0002A5DE6A|URS0002A5FE14|URS0002A630C0|URS0002A64195|URS0002A662CB|URS0002A66EB8|URS0002A6802A|URS0002A68269|URS0002A6878B|URS0002A699EB|URS0002A6B235		
CYP1B1-AS1	ENSG00000232973	CGGA_325	169	285154|28543|C2ORF58|CYP1B1-AS1|CYP1B1-AS1-201|CYP1B1-AS1-202|CYP1B1-AS1-203|CYP1B1-AS1-204|CYP1B1-AS1-205|CYP1B1-AS1-206|CYP1B1-AS1-207|CYP1B1-AS1-208|CYP1B1-AS1-209|CYP1B1-AS1-210|CYP1B1-AS1-211|CYP1B1-AS1-212|CYP1B1-AS1-213|CYP1B1-AS1-214|CYP1B1-AS1-215|CYP1B1-AS1-216|CYP1B1-AS1-217|CYP1B1-AS1-218|CYP1B1-AS1-219|CYP1B1-AS1-220|CYP1B1-AS1-221|CYP1B1-AS1-222|CYP1B1-AS1-223|CYP1B1-AS1-224|CYP1B1-AS1-225|CYP1B1-AS1-226|CYP1B1-AS1-227|CYP1B1-AS1-228|CYP1B1-AS1-229|CYP1B1-AS1-230|CYP1B1-AS1-231|CYP1B1-AS1-232|CYP1B1-AS1-233|CYP1B1-AS1-234|CYP1B1-AS1-235|CYP1B1-AS1-236|CYP1B1-AS1-237|CYP1B1-AS1-238|CYP1B1-AS1-239|CYP1B1-AS1-240|CYP1B1-AS1-241|CYP1B1-AS1-242|CYP1B1-AS1-243|CYP1B1-AS1-244|CYP1B1-AS1-245|CYP1B1-AS1-246|CYP1B1-AS1-247|CYP1B1-AS1-248|CYP1B1-AS1-249|CYP1B1-AS1-250|CYP1B1-AS1-251|CYP1B1-AS1-	CYP1B1-AS1	True

252|CYP1B1-AS1-253|CYP1B1-AS1-254|CYP1B1-AS1-
255|CYP1B1-AS1-256|CYP1B1-AS1-257|CYP1B1-AS1-
258|CYP1B1-AS1-259|CYP1B1-AS1-260|CYP1B1-AS1-
261|CYP1B1-AS1-262|CYP1B1-AS1-263|CYP1B1-AS1-
264|CYP1B1-AS1-265|CYP1B1-AS1-266|CYP1B1-AS1-
267|CYP1B1-AS1-268|CYP1B1-AS1-269|CYP1B1-AS1-
270|CYP1B1-AS1-271|CYP1B1-AS1-272|CYP1B1-AS1-
273|CYP1B1-AS1-274|CYP1B1-AS1-275|CYP1B1-AS1-
276|CYP1B1-AS1-277|CYP1B1-AS1-
278|GO:0000244|GO:0000353|GO:0005688|GO:0030621|
GO:0046540|HGNC:28543|MGC34824|NR_027252|NR_
027252.1|URS000046253D|URS00004723A8|URS00004
C21D3|URS00004C2A84|URS00004FF70A|URS000076
C4F9|URS000078116B|URS00008116DF|URS00008117
C8|URS0000811815|URS0000811920|URS0000811A98|
URS0000811C13|URS0000811D03|URS0000811D18|UR
S0000811D54|URS0000811DBA|URS0000811DE5|URS
0000811F29|URS0000812033|URS0000DB8835|URS000
0EEF01A|URS0000EF552E|URS0000EF6824|URS00022
B3FF8|URS00025E62BF|URS00025F35FB|URS0002A17
B38|URS0002A19198|URS0002A1BF8E|URS0002A253
7B|URS0002A27E89|URS0002A29523|URS0002A297A
2|URS0002A29854|URS0002A2B765|URS0002A2D853|
URS0002A2F09D|URS0002A30A26|URS0002A30ADA|
URS0002A30AEE|URS0002A30F4D|URS0002A3416F|
URS0002A34A81|URS0002A34B20|URS0002A36BE5|U
RS0002A39C62|URS0002A3D24A|URS0002A3D996|U
RS0002A3E21F|URS0002A41557|URS0002A4367E|UR
S0002A44257|URS0002A448A7|URS0002A47699|URS0
002A495A4|URS0002A49981|URS0002A49E4B|URS00
02A4D694|URS0002A4FB2B|URS0002A50437|URS000
2A568D2|URS0002A5ADDB|URS0002A5B64D|URS000
2A5C612|URS0002A5DC06|URS0002A5DD71|URS000
2A5DE6A|URS0002A5FE14|URS0002A630C0|URS0002
A64195|URS0002A662CB|URS0002A66EB8|URS0002A
6802A|URS0002A68269|URS0002A6878B|URS0002A69
9EB|URS0002A6B235

Table S6b.

Hazard ratios per standard deviation of within-cohort expression, TCGA and both CGGA cohorts.

cohort	gene	n	events	HR_perSD	lower	upper	p	logHR	se
TCGA (PFI, discovery)	LINC00707	69	51	1.273	0.9019	1.797	0.1697	0.2415	0.1759
TCGA (PFI, discovery)	ZFPM2-AS1	69	51	1.348	0.9862	1.842	0.06113	0.2985	0.1594
TCGA (PFI, discovery)	MROCKI	69	51	0.5781	0.4098	0.8154	0.001792	-0.548	0.1755
TCGA (PFI, discovery)	LINC01936	69	51	1.221	0.9222	1.618	0.1631	0.2	0.1434
TCGA (PFI, discovery)	AC083864.5	69	51	0.8427	0.6272	1.132	0.2563	-0.1711	0.1507
TCGA (PFI, discovery)	H19	69	51	1.068	0.7776	1.467	0.6841	0.06589	0.1619
TCGA (PFI, discovery)	DNM3OS	69	51	1.136	0.8824	1.461	0.3231	0.1272	0.1287
TCGA (PFI, discovery)	CYP1B1-AS1	69	51	1.276	0.9504	1.713	0.1049	0.2437	0.1503
TCGA (OS, discovery)	LINC00707	69	50	1.723	1.236	2.401	0.001329	0.5439	0.1694
TCGA (OS, discovery)	ZFPM2-AS1	69	50	1.594	1.198	2.121	0.001385	0.4662	0.1458
TCGA (OS, discovery)	MROCKI	69	50	0.4356	0.3001	0.6323	1.24e-05	-0.8309	0.1901
TCGA (OS, discovery)	LINC01936	69	50	1.32	0.9884	1.763	0.06003	0.2775	0.1476
TCGA (OS, discovery)	AC083864.5	69	50	0.8192	0.5966	1.125	0.2179	-0.1994	0.1618

TCGA (OS, discovery)	H19	69	50	1.105	0.7895	1.547	0.56	0.1	0.1717
TCGA (OS, discovery)	DNM3OS	69	50	1.279	1.008	1.622	0.04274	0.2459	0.1214
TCGA (OS, discovery)	CYP1B1-AS1	69	50	1.493	1.097	2.03	0.0107	0.4006	0.157
CGGA_693	LINC00707	76	66	0.9301	0.7266	1.191	0.5649	-0.0725	0.126
CGGA_693	H19	76	66	1.142	0.8918	1.461	0.2932	0.1324	0.126
CGGA_693	CYP1B1-AS1	76	66	0.8339	0.6378	1.09	0.1841	-0.1817	0.1368
CGGA_325	LINC00707	52	43	1.173	0.7864	1.749	0.4347	0.1593	0.2039
CGGA_325	H19	52	43	0.9524	0.683	1.328	0.774	-0.04873	0.1697
CGGA_325	DNM3OS	52	43	1.081	0.7998	1.46	0.6135	0.07753	0.1535
CGGA_325	CYP1B1-AS1	52	43	0.9075	0.6469	1.273	0.5743	-0.09705	0.1727

Table S6c.

CPTAC-3 GBM validation (GDC, STAR/GENCODE v36; n = 188, 141 deaths), age-adjusted Cox on overall survival.

gene	available	n	events	HR_perSD	lower	upper	p	logHR	se
LINC00707	TRUE	188	141	1.152	0.9909	1.34	0.0656	0.1416	0.07692
ZFPM2-AS1	TRUE	188	141	1.12	0.9677	1.295	0.1289	0.1129	0.07437
MROCKI	TRUE	188	141	0.935	0.7912	1.105	0.4299	-0.06722	0.08517
LINC01936	TRUE	188	141	1.049	0.8942	1.23	0.5585	0.04759	0.08134
AC083864.5	TRUE	188	141	0.9544	0.8119	1.122	0.5719	-0.04664	0.0825
H19	TRUE	188	141	1.135	0.9651	1.336	0.1255	0.127	0.08288
DNM3OS	TRUE	188	141	1.068	0.9033	1.263	0.4405	0.06602	0.08559
CYP1B1-AS1	TRUE	188	141	1.119	0.9487	1.32	0.1821	0.1123	0.08418

Table S6d.

Discovery-excluded pooled external estimates for the screen-nominated candidates.

gene	k	HR	lower	upper	p	I2
LINC00707	3	1.095	0.9686	1.237	0.1472	10.31
H19	3	1.109	0.9779	1.257	0.1071	0
CYP1B1-AS1	3	1.013	0.8895	1.153	0.8485	47.7
DNM3OS	2	1.071	0.9252	1.24	0.3577	0
ZFPM2-AS1	1	1.12	0.9677	1.295	0.1289	
MROCKI	1	0.935	0.7912	1.105	0.4299	
LINC01936	1	1.049	0.8942	1.23	0.5585	
AC083864.5	1	0.9544	0.8119	1.122	0.5719	

Table S7a.

Permutation-matched null for ceRNA-pattern triplet counts (B = 500).

lncRNA	n_triplets_tested	observed_hits	recomputed_hits	null_mean	null_sd	null_q95	null_max	emp_p	B
AC046143.1	527	0	0	0.322	2.278	0	27	1	500
DNM3OS	8055	0	0	0.594	2.135	4	18	1	500
H19	80002		92	8.624	21.02	43.1	249	0.02196	500

Table S7b.

Stoichiometry: estimated molecules per cell for lncRNAs, implicated miRNAs and competing target-site pools.

molecule	layer	median_TPM	q90_TPM	molecules_per_cell_median	molecules_per_cell_q90
H19	lncRNA	576.6	4811	115.3	962.1

DNM3OS	lncRNA	33.69	131.8	6.739	26.36
LINC00707	lncRNA	32.98	83.58	6.597	16.72
ZFPM2-AS1	lncRNA	49.34	235.6	9.867	47.13
LINC01936	lncRNA	28.7	59	5.74	11.8
MROCKI	lncRNA	371	1213	74.2	242.6
hsa-miR-20b-5p	miRNA (precursor-level RPM)	88.62	217.2	8.862	21.72
hsa-miR-148b-3p	miRNA (precursor-level RPM)	1087	1502	108.7	150.2
hsa-miR-130a-3p	miRNA (precursor-level RPM)	1518	2821	151.8	282.1
hsa-miR-519d-3p	miRNA (precursor-level RPM)	0	0.336	0	0.0336
hsa-miR-519c-3p	miRNA (precursor-level RPM)	0	0	0	0
target pool of hsa-miR-20b-5p	competing mRNA sites	2.153e+05		4.305e+04	
target pool of hsa-miR-148b-3p	competing mRNA sites	1.695e+05		3.391e+04	
target pool of hsa-miR-130a-3p	competing mRNA sites	2.133e+05		4.266e+04	
target pool of hsa-miR-519d-3p	competing mRNA sites	2.212e+05		4.424e+04	
target pool of hsa-miR-519c-3p	competing mRNA sites	1.707e+05		3.415e+04	

Table S8.

Eighteen widely cited glioma lncRNAs tested on the progression-free interval under MGMT adjustment (14 detectable).

gene	detected	HR	lo	hi	p	fdr
MALAT1	TRUE	1.405	1.049	1.883	0.02278	0.3189
XIST	TRUE	0.4127	0.1496	1.139	0.08743	0.4688
GAS5	TRUE	0.7558	0.5383	1.061	0.1058	0.4688
NEAT1	TRUE	1.256	0.9311	1.695	0.1354	0.4688
HOXA-AS3	TRUE	0.7946	0.5722	1.103	0.1699	0.4688
HOXA-AS2	TRUE	0.8392	0.6195	1.137	0.2578	0.4688
HOTAIR	TRUE	0.8408	0.6151	1.149	0.2765	0.4688
SBF2-AS1	TRUE	0.8397	0.6118	1.153	0.2798	0.4688
DNM3OS	TRUE	1.136	0.8824	1.461	0.3231	0.4688
LINC00461	TRUE	0.8678	0.6505	1.158	0.3349	0.4688
MIR155HG	TRUE	0.8953	0.6714	1.194	0.4513	0.5743
H19	TRUE	1.068	0.7776	1.467	0.6841	0.7981
CRNDE	TRUE	1.028	0.7459	1.416	0.8678	0.895
MEG3	TRUE	0.9796	0.7213	1.33	0.895	0.895
TUG1	FALSE					
HOTTIP	FALSE					
UCA1	FALSE					
LINC-ROR	FALSE					

Table S9.

Endpoint audit: events, censoring, median time, follow-up and six-month event-free rate for each endpoint and cohort.

endpoint	n	events	censored	median_time	median_followup	censored_before_6mo	event_before_6mo	pct_event_free_6mo
'PFS' as originally analyzed (n=94)	94	68	26	15.31	90.93	8	14	84.5
OS rebuilt from GDC (n=94)	94	68	26	15.31	90.93	8	14	84.5
OS, TCGA-CDR (n=94)	94	68	26	15.31	90.93	8	14	84.5
PFI, TCGA-CDR (n=94)	94	71	23	8.41	31.24	11	32	64
OS rebuilt from GDC (MGMT-known n=69)	69	50	19	14.95	31.24	5	14	79.2
PFI, TCGA-CDR (MGMT-known n=69)	69	51	18	7.79	22.77	8	25	62

Table S10.

The 16 transcripts nominated by the unadjusted screen, re-tested on the progression-free interval.

gene_id	gene_name	HR	lower	upper	p	fdr_transcriptome wide	endpoint	old_HR	old_p	old_fdr16	fdr_within16
ENSG00000251003.9	ZFPM2-AS1	1.236	0.9901	1.543	0.06113	0.3303	PFI (primary)	1.392	0.001422	0.01138	0.5125
ENSG00000232973.13	CYP1B1-AS1	1.194	0.9637	1.478	0.1049	0.3669	PFI (primary)	1.346	0.009972	0.03989	0.5125
ENSG00000235997.3	LINC01936	1.26	0.9107	1.743	0.1631	0.4269	PFI (primary)	1.39	0.05371	0.1228	0.5125
ENSG00000238266.2	LINC00707	1.203	0.9239	1.568	0.1697	0.4339	PFI (primary)	1.528	0.001262	0.01138	0.5125
ENSG00000283907.1	AD000090.1	0.8885	0.741	1.065	0.2018	0.4757	PFI (primary)	0.8841	0.2487	0.3317	0.5125
ENSG00000280800.1	FP671120.6	1.113	0.9384	1.319	0.2193	0.4977	PFI (primary)	0.9631	0.7122	0.7597	0.5125
ENSG00000268941.2	LINC01711	1.176	0.9025	1.533	0.23	0.5099	PFI (primary)	1.266	0.1052	0.1871	0.5125
ENSG00000287893.1	AC083864.5	0.8862	0.7194	1.092	0.2563	0.535	PFI (primary)	0.8679	0.2143	0.3117	0.5125
ENSG00000230630.5	DNM3OS	1.089	0.9198	1.289	0.3231	0.589	PFI (primary)	1.18	0.04273	0.114	0.5331
ENSG00000228536.3	LYPLAL1-AS1	1.107	0.9008	1.361	0.3332	0.598	PFI (primary)	1.251	0.02268	0.07258	0.5331
ENSG00000229334.1	AC046143.1	1.154	0.7608	1.751	0.5002	0.7214	PFI (primary)	1.786	0.002748	0.01466	0.7276
ENSG00000231427.6	LINC01445	0.9529	0.7822	1.161	0.6319	0.8097	PFI (primary)	1.047	0.6704	0.7597	0.842
ENSG00000130600.19	H19	1.023	0.9155	1.144	0.6841	0.8399	PFI (primary)	1.039	0.5304	0.6528	0.842
ENSG00000228203.7	GRASLND	0.9642	0.743	1.251	0.7839	0.8876	PFI (primary)	1.233	0.08616	0.1723	0.8959
ENSG00000287064.1	AL606500.1	1.017	0.8405	1.231	0.8609	0.928	PFI (primary)	0.9882	0.908	0.908	0.9183
ENSG00000253161.5	LINC01605	0.9927	0.7746	1.272	0.954	0.9794	PFI (primary)	1.172	0.2067	0.3117	0.954

Table S11.

Bootstrap optimism correction of the C-index, with and without repeating candidate selection inside the resample. Selection-inclusive rows (all 3,785 lncRNAs re-ranked and the top-ranked transcript re-selected within every resample) are reported for both the PFI and OS endpoints; all other rows treat the named transcript as pre-specified, i.e. without repeating the selection step.

analysis	gene	C_baseline	C_full	dC_apparent	optimism	dC_corrected	optimism_lo	optimism_hi	B
selection repeated inside bootstrap (top-1 of 3785)	ENSG00000286342.1	0.5386	0.645	0.1064	0.04989	0.05649	-0.04616	0.1464	300
LINC00707 (pre-specified)	LINC00707	0.5386	0.5784	0.03975	0.004551	0.0352	-0.05308	0.09262	300
ZFPM2-AS1 (pre-specified)	ZFPM2-AS1	0.5386	0.589	0.05039	-0.003932	0.05432	-0.06132	0.06013	300
OS: LINC00707 (pre-specified)	LINC00707	0.5664	0.6794	0.1129	-0.0103	0.1232	-0.08847	0.08458	300
OS: ZFPM2-AS1 (pre-specified)	ZFPM2-AS1	0.5664	0.6675	0.1011	-0.01308	0.1141	-0.07902	0.06085	300
OS: MROCKI (pre-specified)	MROCKI	0.5664	0.6937	0.1273	-0.02244	0.1497	-0.1179	0.06351	300
OS: selection repeated inside bootstrap (top-1 of 3785)	MROCKI	0.5664	0.6937	0.1273	0.056	0.071	NA	NA	300

Table S12.

Joint TCGA + CPTAC-3 transcriptome-wide meta-analysis on overall survival (top rows; full table in the accompanying file).

Showing the first 40 of 3784 rows; full table: joint_discovery_tcga_cptac_OS.tsv

gene_id	logHR_tcga	se_tcga	p_tcga	logHR_cptac	se_cptac	p_cptac	w1	w2	logHR_meta	se_meta	p_meta	fdr_meta	concorda
ENSG00000258498.8	0.427	0.1311	0.001124	0.2769	0.08396	9.73e-04	58.19	141.9	0.3206	0.0707	5.78e-06	0.02188	TRUE
ENSG00000180539.8	0.5191	0.1406	2.23e-04	0.222	0.08724	0.01095	50.56	131.4	0.3045	0.07413	3.99e-05	0.06529	TRUE
ENSG00000257337.7	0.4296	0.137	0.001713	0.2488	0.08925	0.005308	53.28	125.6	0.3027	0.07478	5.18e-05	0.06529	TRUE
ENSG00000287090.1	-0.326	0.1364	0.01685	-0.2424	0.08503	0.004354	53.76	138.3	-0.2658	0.07215	2.30e-04	0.1821	TRUE
ENSG00000227392.2	0.2902	0.1276	0.02296	0.2303	0.08074	0.004346	61.39	153.4	0.2474	0.06823	2.88e-04	0.1821	TRUE
ENSG00000176593.8	-0.2313	0.1144	0.04317	-0.2576	0.08547	0.002574	76.41	136.9	-0.2482	0.06847	2.89e-04	0.1821	TRUE
ENSG00000231616.9	-0.02502	0.1439	0.862	-0.3237	0.08566	1.58e-04	48.28	136.3	-0.2455	0.07361	8.51e-04	0.3559	TRUE
ENSG00000286970.1	0.1315	0.13	0.312	0.284	0.0858	9.34e-04	59.14	135.9	0.2377	0.07161	9.02e-04	0.3559	TRUE
ENSG00000287129.1	-0.04997	0.1346	0.7104	-0.2975	0.0815	2.62e-04	55.23	150.6	-0.231	0.06971	9.19e-04	0.3559	TRUE
ENSG00000228288.7	0.1057	0.1326	0.4252	0.2657	0.07926	8.00e-04	56.88	159.2	0.2236	0.06803	0.001013	0.3559	TRUE
ENSG00000287706.1	-0.4029	0.1431	0.004869	-0.1874	0.08859	0.03437	48.83	127.4	-0.2471	0.07533	0.001035	0.3559	TRUE
ENSG00000267272.5	0.2578	0.1533	0.09258	0.2407	0.08795	0.006197	42.56	129.3	0.245	0.07628	0.001322	0.3926	TRUE
ENSG00000271086.7	-0.005274	0.139	0.9697	-0.3076	0.08292	2.07e-04	51.78	145.4	-0.2283	0.07121	0.001349	0.3926	TRUE
ENSG00000238287.1	0.2037	0.1314	0.1212	0.2328	0.08631	0.006999	57.87	134.2	0.224	0.07215	0.001903	0.4316	TRUE
ENSG00000288011.1	0.3922	0.143	0.006076	0.1684	0.08572	0.0495	48.93	136.1	0.2276	0.07351	0.001964	0.4316	TRUE
ENSG00000249700.9	0.3101	0.1288	0.01606	0.1681	0.07817	0.03151	60.27	163.7	0.2063	0.06682	0.002018	0.4316	TRUE
ENSG00000222012.1	0.3259	0.1313	0.01303	0.1757	0.08595	0.04098	58.03	135.4	0.2208	0.07191	0.002141	0.4316	TRUE
ENSG00000256546.2	0.04115	0.113	0.7158	0.3269	0.09031	2.95e-04	78.29	122.6	0.2155	0.07055	0.002252	0.4316	TRUE
ENSG00000234222.6	0.2959	0.1399	0.03444	0.1916	0.0836	0.02191	51.08	143.1	0.219	0.07177	0.002272	0.4316	TRUE
ENSG00000286615.1	0.3797	0.1296	0.003384	0.1444	0.08313	0.08241	59.57	144.7	0.213	0.06997	0.002332	0.4316	TRUE
ENSG00000280434.1	0.1481	0.1273	0.2445	0.2463	0.08562	0.004021	61.75	136.4	0.2157	0.07104	0.002395	0.4316	TRUE
ENSG00000248714.7	0.2884	0.1379	0.03642	0.174	0.07621	0.02245	52.61	172.2	0.2008	0.0667	0.002612	0.4411	TRUE

ENSG00000174403.16	0.1603	0.1273	0.2082	0.2178	0.0798	0.006353	61.66	157	0.2016	0.06762	0.002876	0.4411	TRUE
ENSG00000227502.3	-0.7185	0.1545	3.29e-06	-0.06722	0.08517	0.4299	41.91	137.9	-0.2191	0.07458	0.003312	0.4411	TRUE
ENSG00000272070.1	-0.4432	0.1446	0.002168	-0.1386	0.0893	0.1207	47.85	125.4	-0.2227	0.07598	0.003372	0.4411	TRUE
ENSG00000232104.3	-0.1876	0.1406	0.1818	-0.2209	0.08574	0.01	50.62	136	-0.2118	0.0732	0.003802	0.4411	TRUE
ENSG00000224596.8	0.2794	0.1238	0.02407	0.1544	0.07747	0.04633	65.22	166.6	0.1895	0.06568	0.003906	0.4411	TRUE
ENSG00000269950.1	-0.0679	0.1349	0.6147	-0.259	0.084	0.002052	54.96	141.7	-0.2056	0.07131	0.003942	0.4411	TRUE
ENSG00000260788.6	0.2456	0.1223	0.04468	0.1795	0.08487	0.03443	66.84	138.8	0.201	0.06973	0.003949	0.4411	TRUE
ENSG00000260000.2	-0.282	0.1328	0.03367	-0.1798	0.08789	0.04075	56.72	129.5	-0.211	0.07329	0.003996	0.4411	TRUE
ENSG00000272732.1	-0.09327	0.1182	0.4303	-0.2593	0.08679	0.00281	71.52	132.7	-0.2012	0.06997	0.004037	0.4411	TRUE
ENSG00000284747.1	0.2719	0.1429	0.05715	0.1952	0.08856	0.02754	48.94	127.5	0.2164	0.07528	0.004039	0.4411	TRUE
ENSG00000257060.7	-0.2463	0.1231	0.04543	-0.1837	0.0874	0.0356	65.97	130.9	-0.2047	0.07127	0.004082	0.4411	TRUE
ENSG00000237410.1	0.4096	0.1303	0.001665	0.1182	0.09004	0.1891	58.92	123.4	0.2124	0.07407	0.004132	0.4411	TRUE
ENSG00000255737.2	0.1424	0.1305	0.2749	0.2253	0.08339	0.006891	58.76	143.8	0.2013	0.07026	0.004173	0.4411	TRUE
ENSG00000286786.1	-0.1769	0.1407	0.2086	-0.2236	0.08656	0.009777	50.51	133.5	-0.2108	0.07372	0.004245	0.4411	TRUE
ENSG00000225756.1	0.3207	0.1278	0.01209	0.1472	0.08296	0.07606	61.22	145.3	0.1986	0.06959	0.004313	0.4411	TRUE
ENSG00000272084.1	0.1202	0.1274	0.3454	0.2368	0.08487	0.005267	61.61	138.8	0.201	0.07063	0.004437	0.4418	TRUE
ENSG00000231672.8	0.08304	0.1292	0.5205	0.264	0.08828	0.002787	59.89	128.3	0.2064	0.07289	0.004632	0.4494	TRUE
ENSG00000225140.1	0.2225	0.1224	0.06922	0.1663	0.07597	0.02857	66.71	173.3	0.1819	0.06455	0.004828	0.4568	TRUE

Table S13.

DIO3OS and C9orf139: stress testing within TCGA against MGMT status, cellular composition and endpoint.

gene	model	n	events	HR	lo	hi	p	zph_p
DIO3OS	OS, age only (n=69)	69	50	1.491	1.117	1.99	0.006649	0.2732
DIO3OS	OS, MGMT-adjusted	69	50	1.438	1.048	1.974	0.0244	0.2849
DIO3OS	OS, MGMT+composition	69	50	1.764	1.21	2.57	0.003165	0.2235
DIO3OS	PFI, MGMT-adjusted	69	51	1.167	0.8698	1.567	0.3027	0.2265
DIO3OS	PFI, MGMT+composition	69	51	1.309	0.9148	1.874	0.1406	0.1967
C9orf139	OS, age only (n=69)	69	50	1.588	1.156	2.183	0.004367	0.06488
C9orf139	OS, MGMT-adjusted	69	50	1.58	1.176	2.123	0.002407	0.1894
C9orf139	OS, MGMT+composition	69	50	1.738	1.219	2.476	0.002242	0.2786
C9orf139	PFI, MGMT-adjusted	69	51	1.267	0.9608	1.672	0.09355	0.6493
C9orf139	PFI, MGMT+composition	69	51	1.339	0.9783	1.833	0.06829	0.7507
AC068888.1	OS, age only (n=69)	69	50	1.414	1.038	1.928	0.02816	0.04912
AC068888.1	OS, MGMT-adjusted	69	50	1.336	0.9701	1.839	0.07605	0.05525
AC068888.1	OS, MGMT+composition	69	50	1.348	0.9645	1.884	0.08037	0.05507
AC068888.1	PFI, MGMT-adjusted	69	51	0.8458	0.5983	1.196	0.343	0.5658
AC068888.1	PFI, MGMT+composition	69	51	0.8288	0.5749	1.195	0.3142	0.4969

Table S14.

DIO3OS and C9orf139 in the held-out CGGA cohorts.

cohort	gene	annotated	n	events	HR	lo	hi	p
CGGA_693	DIO3OS	TRUE	76	66	0.8584	0.6576	1.12	0.2613
CGGA_693	C9orf139	TRUE	76	66	0.8315	0.5993	1.154	0.2696
CGGA_693	AC068888.1	FALSE						
CGGA_325	DIO3OS	TRUE	52	43	0.9535	0.6692	1.358	0.7919
CGGA_325	C9orf139	TRUE	52	43	0.8559	0.6067	1.207	0.3755

CGGA_325 AC068888.1 FALSE

Table S15.

DLK1-DIO3 imprinted locus, gene by gene, in both cohorts.

gene	in_TCGA	in_CPTAC	HR_tcga	p_tcga	HR_cptac	p_cptac
DIO3OS	TRUE	TRUE	1.438	0.0244	1.319	9.73e-04
MEG3	TRUE	TRUE	0.9214	0.6427	1.07	0.4629
MEG8	TRUE	TRUE	1.038	0.8029	1.025	0.7885
MEG9	TRUE	TRUE	0.9925	0.9639	1.074	0.4279
RTL1	FALSE	FALSE				
DLK1	FALSE	FALSE				
MIR381HG	FALSE	TRUE			1.096	0.3131

Table S16.

Bootstrap-corrected delta-C-index for DIO3OS and C9orf139 over the MGMT + clinical baseline.

gene	C_base	C_full	dC_apparent	optimism	dC_corrected
DIO3OS	0.5664	0.6326	0.06613	-0.004979	0.0711
C9orf139	0.5664	0.6687	0.1023	-0.01043	0.1127

Table S17.

TCGA-GBM treatment-group assessment: temozolomide-treated, untreated and unclassifiable cases with RNA-seq.

Showing the first 40 of 284 rows; full table: tcga_treatment_groups.tsv

sid	file_id	grp	idh	mgmt	vital	os_days	tmz
TCGA-06-6390	d4649bc7-09d2-4d90-bafd-0bec72429b00	TMZ	WT	UNMETHYLATED	Dead	164	True
TCGA-06-5411	77c261f0-384c-4906-a57e-7c3b35c17041	pharma_unspecified	WT	UNMETHYLATED	Dead	254	False
TCGA-12-3648	afbd1975-263b-404b-a738-886bf25c0223	pharma_unspecified	WT	METHYLATED	Dead	819	False
TCGA-06-A7TK	444a2332-9e52-4cf8-94d9-07a8b829633e	TMZ			Dead	444	True
TCGA-19-5959	07c46064-9fb9-4aad-9289-187d156c9d27	NO_pharma	WT	METHYLATED	Dead	511	False
TCGA-06-0166	b7a15790-9999-4fd2-b327-9584e1445c1f	NO_pharma	WT		Dead	178	False
TCGA-06-5859	9813657d-da8b-4b27-9815-4b7d7b4bbac4	TMZ	WT	UNMETHYLATED	Alive	139	True
TCGA-14-4157	2bac85df-4900-41cd-a2ae-e33db7abd9d1	NO_pharma	R132H	METHYLATED	Alive		False
TCGA-12-5299	a7260181-c266-427f-abba-4aa318f1710c	TMZ	WT	UNMETHYLATED	Dead	98	True
TCGA-06-5416	072f2d4e-db08-40c2-891a-9199e47fcac3	TMZ		UNMETHYLATED	Alive	204	True
TCGA-19-1390	e676c661-cae3-4755-a90f-e632a3d82850	NO_pharma	WT	METHYLATED	Dead	772	False
TCGA-16-0846	cf92132f-edaf-47dc-9469-69f3cef16737	NO_pharma	WT	METHYLATED	Dead	119	False
TCGA-02-0047	6835ae68-2f18-4239-ac8c-6a8291101e45	NO_pharma	WT	UNMETHYLATED	Dead	448	False
TCGA-14-0817	b7196540-a319-41a0-befd-c363e511ac1d	no_record	WT	UNMETHYLATED	Dead	164	False
TCGA-26-5136	1b8cc228-a020-4418-be03-ad409d781728	no_record	WT	METHYLATED	Dead	577	False
TCGA-28-2514	7a7637d0-110f-4809-a79f-9eead03c762b	TMZ	WT	UNMETHYLATED	Alive	160	True
TCGA-19-2620	f2eb8d3a-ee6e-4180-921d-0ef8f7d1ead5	TMZ	WT	METHYLATED	Dead	148	True
TCGA-12-3650	dbcf47dd-1c8d-4736-9ea2-08c03a94b298	pharma_unspecified	WT	UNMETHYLATED	Dead	333	False
TCGA-12-0772	9d938732-9077-4d77-ba5b-e8354babf909	pharma_unspecified			Dead	1638	False
TCGA-06-2569	c96554bf-ae42-4ec0-93fd-7c2ab01d19f0	no_record	WT	UNMETHYLATED	Alive	13	False
TCGA-06-0644	03fc636b-9e3d-42ea-88cf-8081539a9859	pharma_unspecified	WT		Dead	384	False
TCGA-41-4097	3449e073-b0f2-4498-b9db-18e8593ef580	NO_pharma	WT	UNMETHYLATED	Dead	6	False
TCGA-26-5135	bec610b2-9082-429d-8aef-6cbf3bb7a020	no_record	WT	METHYLATED	Dead	270	False

TCGA-32-1970	69f7dc91-32d1-4359-850f-860aab36e433	NO_pharma	WT	UNMETHYLATED	Dead	468	False
TCGA-28-2510	c5edc8d9-bae8-4841-802c-919dc0d4c055	no_record					False
TCGA-41-2571	f982722f-ae1d-4b7a-b445-0646517acb0f	TMZ	WT	UNMETHYLATED	Dead	26	True
TCGA-14-0871	9240b811-4132-4ca4-b44e-7e672b594657	no_record	WT	UNMETHYLATED	Dead	880	False
TCGA-28-5213	155e74da-ebd4-4f87-ac74-7bf9fd74644f	TMZ	WT	UNMETHYLATED	Alive	951	True
TCGA-27-1834	9ac00a37-7f08-4c9f-ab77-4019a05c5083	TMZ	WT	METHYLATED	Dead	1233	True
TCGA-12-0775	54bbc769-1001-4bdc-991a-e1ef0b969aef	TMZ	WT		Dead	232	True
TCGA-06-5414	1bc3c302-0e49-4944-b3ce-7d6e6f846493	NO_pharma	WT	UNMETHYLATED	Alive		False
TCGA-06-0190	ba9afa8e-405c-49b7-bc66-8ab1af1f942e	pharma_unspecified	WT		Dead	317	False
TCGA-06-6388	d6278bca-8b01-4761-8457-6a67c63c78b1	pharma_unspecified	WT		Dead	159	False
TCGA-02-2486	f241eee0-28ea-4700-ba91-d3d50c2f7e48	TMZ	WT	UNMETHYLATED	Dead	618	True
TCGA-14-0790	689956e8-e5b8-4d66-a83d-9fc0c82fc2c6	TMZ	WT	METHYLATED	Dead	419	True
TCGA-06-6694	90bb68a5-2311-4e19-80d4-35bf447456cd	no_record			Dead	224	False
TCGA-12-1095	2e4989d4-fde3-4da7-b656-5e07dcf28b14	pharma_unspecified		UNMETHYLATED	Dead	482	False
TCGA-06-5410	9a3e22cb-36b6-43ae-8f5c-b991f32c4732	NO_pharma	WT	METHYLATED	Dead	108	False
TCGA-76-4926	b4abde15-98d3-4e78-9170-6c372de5c0f3	pharma_unspecified	WT	UNMETHYLATED	Dead	138	False
TCGA-19-5960	4bc70225-3511-4571-8029-7ac499e60742	TMZ	WT	UNMETHYLATED	Dead	455	True

Table S18.

Numbers at risk and cumulative events for the median-split survival curves of Figure 5, with the pre-specified variance-stabilized expression cutpoint used in each cohort.

gene	cohort	cutpoint_VST	group	time_months	n_at_risk	n_events_cum
DIO3OS	TCGA (n = 94)	4.63	Low	0	47	0
DIO3OS	TCGA (n = 94)	4.63	Low	6	37	5
DIO3OS	TCGA (n = 94)	4.63	Low	12	32	1
DIO3OS	TCGA (n = 94)	4.63	Low	18	19	10
DIO3OS	TCGA (n = 94)	4.63	Low	24	12	7
DIO3OS	TCGA (n = 94)	4.63	Low	36	5	5
DIO3OS	TCGA (n = 94)	4.63	Low	48	0	5
DIO3OS	TCGA (n = 94)	4.63	Low	60	0	0
DIO3OS	TCGA (n = 94)	4.63	High	0	47	0
DIO3OS	TCGA (n = 94)	4.63	High	6	35	9
DIO3OS	TCGA (n = 94)	4.63	High	12	21	8
DIO3OS	TCGA (n = 94)	4.63	High	18	11	9
DIO3OS	TCGA (n = 94)	4.63	High	24	7	3
DIO3OS	TCGA (n = 94)	4.63	High	36	3	4
DIO3OS	TCGA (n = 94)	4.63	High	48	3	0
DIO3OS	TCGA (n = 94)	4.63	High	60	3	0
DIO3OS	CPTAC-3 (n = 188)	4.1	Low	0	94	0
DIO3OS	CPTAC-3 (n = 188)	4.1	Low	6	79	13
DIO3OS	CPTAC-3 (n = 188)	4.1	Low	12	63	13
DIO3OS	CPTAC-3 (n = 188)	4.1	Low	18	42	17
DIO3OS	CPTAC-3 (n = 188)	4.1	Low	24	30	10
DIO3OS	CPTAC-3 (n =	4.1	Low	36	16	7

	188)					
DIO3OS	CPTAC-3 (n = 188)	4.1	Low	48	6	4
DIO3OS	CPTAC-3 (n = 188)	4.1	Low	60	2	2
DIO3OS	CPTAC-3 (n = 188)	4.1	High	0	94	0
DIO3OS	CPTAC-3 (n = 188)	4.1	High	6	73	20
DIO3OS	CPTAC-3 (n = 188)	4.1	High	12	46	22
DIO3OS	CPTAC-3 (n = 188)	4.1	High	18	30	13
DIO3OS	CPTAC-3 (n = 188)	4.1	High	24	22	6
DIO3OS	CPTAC-3 (n = 188)	4.1	High	36	6	12
DIO3OS	CPTAC-3 (n = 188)	4.1	High	48	2	1
DIO3OS	CPTAC-3 (n = 188)	4.1	High	60	0	1
C9orf139	TCGA (n = 94)	6.39	Low	0	47	0
C9orf139	TCGA (n = 94)	6.39	Low	6	37	5
C9orf139	TCGA (n = 94)	6.39	Low	12	30	2
C9orf139	TCGA (n = 94)	6.39	Low	18	19	8
C9orf139	TCGA (n = 94)	6.39	Low	24	10	8
C9orf139	TCGA (n = 94)	6.39	Low	36	6	2
C9orf139	TCGA (n = 94)	6.39	Low	48	3	3
C9orf139	TCGA (n = 94)	6.39	Low	60	3	0
C9orf139	TCGA (n = 94)	6.39	High	0	47	0
C9orf139	TCGA (n = 94)	6.39	High	6	35	9
C9orf139	TCGA (n = 94)	6.39	High	12	23	7
C9orf139	TCGA (n = 94)	6.39	High	18	11	11
C9orf139	TCGA (n = 94)	6.39	High	24	9	2
C9orf139	TCGA (n = 94)	6.39	High	36	2	7
C9orf139	TCGA (n = 94)	6.39	High	48	0	2
C9orf139	TCGA (n = 94)	6.39	High	60	0	0
C9orf139	CPTAC-3 (n = 188)	6.16	Low	0	94	0
C9orf139	CPTAC-3 (n = 188)	6.16	Low	6	77	14
C9orf139	CPTAC-3 (n = 188)	6.16	Low	12	58	17
C9orf139	CPTAC-3 (n = 188)	6.16	Low	18	37	16
C9orf139	CPTAC-3 (n = 188)	6.16	Low	24	28	6
C9orf139	CPTAC-3 (n = 188)	6.16	Low	36	12	8
C9orf139	CPTAC-3 (n = 188)	6.16	Low	48	4	2

C9orf139	CPTAC-3 (n = 188)	6.16	Low	60	1	0
C9orf139	CPTAC-3 (n = 188)	6.16	High	0	94	0
C9orf139	CPTAC-3 (n = 188)	6.16	High	6	75	19
C9orf139	CPTAC-3 (n = 188)	6.16	High	12	51	18
C9orf139	CPTAC-3 (n = 188)	6.16	High	18	35	14
C9orf139	CPTAC-3 (n = 188)	6.16	High	24	24	10
C9orf139	CPTAC-3 (n = 188)	6.16	High	36	10	11
C9orf139	CPTAC-3 (n = 188)	6.16	High	48	4	3
C9orf139	CPTAC-3 (n = 188)	6.16	High	60	1	3
